# Supplementary material for: A high-throughput multiplexing and selection strategy to complete bacterial genomes
Source: Gigascience. 2021 Dec 9;10(12):giab079. doi: 10.1093/gigascience/giab079 (PMC8673558; doi:10.1093/gigascience/giab079)
Supplement: giab079_GIGA-D-21-00176_Revision_1 [file giab079_giga-d-21-00176_revision_1.pdf]

GigaScience

A high-throughput multiplexing and selection strategy to complete bacterial genomes

--Manuscript Draft--

**Manuscript Number:**	GIGA-D-21-00176R1
**Full Title:**	A high-throughput multiplexing and selection strategy to complete bacterial genomes
**Article Type:**	Research
**Funding Information:**	h2020 marie skłodowska-curie actions (801133) — Dr. Sergio Arredondo-Alonso, Dr. Anna K. Pöntinen
	trond mohn foundation (TMS2019TMT04) — Dr. Anna K. Pöntinen, Dr. Rebecca A. Gladstone, Prof. Pål J. Johnsen, Prof. Ørjan Samuelsen, Prof. Jukka Corander
	h2020 european research council (742158) — Prof. Jukka Corander
	joint programming initiative on antimicrobial resistance (JPIAMR2016-AC16/00039) — Dr. Anita C. Schürch

Funding	Recipients
h2020 marie skłodowska-curie actions (801133)	Dr. Sergio Arredondo-Alonso<br>Dr. Anna K. Pöntinen
trond mohn foundation (TMS2019TMT04)	Dr. Anna K. Pöntinen<br>Dr. Rebecca A. Gladstone<br>Prof. Pål J. Johnsen<br>Prof. Ørjan Samuelsen<br>Prof. Jukka Corander
h2020 european research council (742158)	Prof. Jukka Corander
joint programming initiative on antimicrobial resistance (JPIAMR2016-AC16/00039)	Dr. Anita C. Schürch

Abstract:

Background

Bacterial whole-genome sequencing based on short-read sequencing data often results in a draft assembly formed by contiguous sequences. The introduction of long-read sequencing technologies permits to unambiguously bridge those contiguous sequences into complete genomes. However, the elevated costs associated with long-read sequencing frequently limit the number of bacterial isolates that can be long-read sequenced.

Here we evaluated the recently released 96 barcoding kit from Oxford Nanopore Technologies (ONT) to generate complete genomes on a high-throughput basis. In addition, we propose a long-read isolate selection strategy that optimizes a representative selection of isolates from large-scale bacterial collections.

Results

Despite an uneven distribution of long-reads per barcode, near-complete chromosomal sequences (assembly contiguity = 0.89) were generated for 96 Escherichia coli isolates with associated short-read sequencing data. The assembly contiguity of the plasmid replicons was even higher (0.98) which indicated the suitability of the multiplexing strategy for studies focused on resolving plasmid sequences. We benchmarked hybrid and ONT-only assemblies and showed that the combination of ONT sequencing data with short-read sequencing data is still highly desirable: (i) to perform an unbiased selection of isolates for long-read sequencing, (ii) to achieve an optimal genome accuracy and completeness, and (iii) to include small plasmids underrepresented in the ONT library.

Conclusions

The proposed long-read isolate selection ensures completing bacterial genomes of isolates that span the genome diversity inherent in large collections of bacterial isolates. We show the potential of using this multiplexing approach to close bacterial genomes on a high-throughput basis.

**Corresponding Author:**	Sergio Arredondo-Alonso, Ph.D.<br>University of Oslo: Universitetet i Oslo<br>Oslo, NORWAY
**Corresponding Author Secondary Information:**	

Powered by Editorial Manager® and ProduXion Manager® from Aries Systems Corporation

**Corresponding Author's Institution:**	University of Oslo: Universitetet i Oslo
**Corresponding Author's Secondary Institution:**	
**First Author:**	Sergio Arredondo-Alonso, Ph.D.
**First Author Secondary Information:**	
**Order of Authors:**	Sergio Arredondo-Alonso, Ph.D.
	Anna K. Pöntinen
	François Cléon
	Rebecca A. Gladstone
	Anita C. Schürch
	Pål J. Johnsen
	Ørjan Samuelsen
	Jukka Corander
**Order of Authors Secondary Information:**	
**Response to Reviewers:**	We thank the reviewers for their excellent comments and suggestions that have substantially increased the quality and clarity of the new revised version of the manuscript. In our response, we refer to the sections and page numbers from the track changes version of the manuscript.<br><br>Reviewer #1: In their manuscript, Arredondo-Alonso et al. provide an evaluation of the new 96 barcode kit from Oxford Nanopore, as well as some new methods to select a representative set of isolates for long-read sequencing based on preexisting short-read data. The paper is well-written and clear and the isolate selection method is interesting; the evaluation as well as the underlying dataset itself (which could be used for e.g. further methods development or benchmarking) will be of interest to the long-read sequencing community.<br><br>We have, however, a few remarks that we would recommend be addressed prior to the publication of the manuscript:<br><br>Major:<br><br>Q: If short-read data are already available, it is not clear that long-read barcoding is still necessary (as opposed to sequencing a barcode-less pool of high molecular weight DNA from the samples of interest, see https://genomebiology.biomedcentral.com/articles/10.1186/s13059-020-01974-9). This should at least be discussed - of course it would be interesting to see the performance of Ultraplexing on the generated dataset (simply ignoring the information present in the barcodes), but perhaps this is beyond the scope of this manuscript.<br><br>R: Thanks for pointing us to this excellent method to assign non-barcoded ONT sequences using preexisting short-read sequencing data. Unfortunately, we were unsuccessful in trying the tool on our set of 96 isolates due to the memory requirements of Ultraplexing (70 CPU hours and 175 GB of memory estimated for 48 samples). In our case, these requirements were even larger since we are dealing with 96 samples. We created a Singularity image to avoid installing a Perl dependency that could not be installed in our compiler version. However, despite trying Ultraplexer on two different HPC clusters, we could not allocate such a large amount of memory to the Singularity image that we created to run Ultraplexing.<br><br>Since comparing both methods was beyond the scope of the manuscript, which was mainly to benchmark the 96 barcoding kit from ONT, we decided only to discuss the potential pros/cons of using Ultraplexing in the Discussion of the manuscript (page 17).<br><br>We believe the method is fascinating and argue that this alternative can be attractive to reduce even more the ONT cost per isolate due to skipping the long-read barcoding

Powered by Editorial Manager® and ProduXion Manager® from Aries Systems Corporation

step, especially if future releases of Ultraplexing permit to reduce the computational requirements.

Q: Evaluation of the long-read sample selection method. It is not entirely clear whether the method was applied to the collection collection of 3254 isolates or to a subset of 1085 isolates, and whether the number of centroids was set to 1085, or to 96 ("the number of centroids of the selection procedure were set to a large number of desired long-read isolates (n = 1085)"... but the number of centroids correspondes to the number of samples selected for long-read data generation, and actually sequenced were only 96 isolates?).

R: We thank the reviewers for pointing this out. We acknowledge this was not sufficiently clearly described in the main manuscript.

We applied the selection procedure twice on the collection of 3254 isolates:

1) In the Results section 'An isolate selection spanning the genome diversity inherent in a short-read collection', we show the potential of the selection approach by just selecting 96 isolates (96 centroids). This was an arbitrary number that we selected because it matched with the maximum number of isolates that can be simultaneously multiplexed in the same ONT flow cell. These 96 hypothetical selected isolates have never been long-read sequenced and thus are different from those presented in the later Results section.

2) As part of an ongoing project, we selected 1085 isolates from the collection of 3254 E. coli isolates using the selection approach described in the Results section 'An isolate selection spanning the genome diversity inherent in a short-read collection'. From these 1085 selected isolates, we investigated different possibilities to complete all these genomes in a high-throughput manner. In the manuscript, we show the results of multiplexing 96 from these 1085 isolates in the same MinION flow cell. These 96 isolates are different from those hypothetically selected at the Results section 'A long-read selection spanning the genome diversity inherent in a short-read collection', since they were selected using a different number of centroids (96 vs 1085).

We have explicitly clarified this difference at the end of the Results section 'An isolate selection spanning the genome diversity inherent in a short-read collection' (pages 10, 11) and the Methods section 'Collection of Illumina short-read assemblies' (pages 6, 7).

Q: Furthermore, the authors report that their selection of isolates covers 99.9% of variation present in the tsne matrix - this is a relevant statistic, but it would be important to complement this with additional statistics on how well their selection captures variation in the underlying matrix of orthologous genes - e.g. it would be important to know how many (of the total considered set) orthologous genes are present in the selected set, potentially
broken down by frequency (e.g. showing a histogram over orthologous gene frequency on the X axis, and the proportion of genes in the corresponding bin represented in the selected set shown as an additional metric on the Y axis for each bin).

R: This is an outstanding comment. We have followed the suggestion of the reviewers of creating a histogram showing the frequency of the orthologous genes grouping the genes in bins (based on their relative frequency). For each bin (n = 100), we have coloured the proportion of genes of that bin that were selected in the 96 isolates. This histogram is available as the new Supplementary Figure S1. From this figure, it is clear that the 96 isolates cover all orthologous genes which are present at a frequency higher than 0.05 (from 4th to 100th bin) and only for those genes present at a frequency of 0.01 (first bin in the histogram) there is a significant proportion of genes not selected. The genes present at a frequency of 0.01 most likely correspond to either phage related genes or genes present in particular extrachromosomal elements which are not shared by all the isolates from a particular k-means cluster.

These new confirmatory results are now summarised in the Results section 'An isolate selection spanning the genome diversity inherent in a short-read collection' (page 10).

Powered by Editorial Manager® and ProduXion Manager® from Aries Systems Corporation

Q: Also, please provide explicit formulae for between_SS and tot_SS.

R: We have provided an explicit formula for between_SS and tot_SS in the Methods section ‘Isolate selection based on existing short-read assemblies’ (page 5).

Q: The sequencing here was carried out with FLO-MIN106 (R9.4) flow cells. The base pair accuracy of Nanopore-only assemblies based on data generated with FLO-MIN111 (R10) flow cells would likely have been higher. This should be mentioned e.g. in the Discussion.

R: Thanks for pointing this out. We have added this excellent remark in the Discussion (page 17).

Q: For the accuracy of the Nanopore-only assemblies: Empirically we have found that running multiple rounds of Medaka polishing can improve the quality of the assemblies. Can the authors check on a subset of samples that this is not the case for the assemblies reported on here, or, if it is the case, also evaluate the accuracy of these assemblies after two rounds of polishing?

R: Thanks for this excellent comment. Following the suggestion, we performed a second round of Medaka polishing on all the genomes, and evaluated their accuracy in terms of SNPs, indels, interrupted ORFs and recovered BUSCO genes. For all metrics, the results were highly similar to the genomes obtained after a single round of Medaka polishing. We have included these new results in the Results section ‘Genome accuracy and completeness of hybrid and ONT-only assemblies’ (pages 14,15).

Minor:

Q: Figure 5: In the legend, A and B are switched (in the figure itself, panel A shows SNPs and panel B INDELs). The authors could consider showing SNPs and INDELs on the log scale, as the differences in achieved accuracy between Flye-only, Flye+Medaka and Unicycler are hard to discern for higher coverages. The authors could also consider combining the Flye-only, Flye+Medaka and Unicycler plots (perhaps connecting the dots corresponding the same isolate with lines and using color to indicate assembly method) -- this type of visualization may give an even better idea of the per-isolate differences in accuracy.

R: We thank the reviewers for spotting the error in the legend of Figure 5, we have now addressed this (page 22).

Following the suggestions of the reviewers, we have decided to redraw Figure 5 in which we now compare SNPs and indels on a log scale, connecting with a dashed line the observations from the same isolate and using colors to differentiate the assemblers. For the number of early interrupted ORFs and recovered BUSCO genes, we have plotted the observations in the same manner but conserving their natural scale. This new visualization is excellent to observe that the number of SNPs is similar between ONT-only and hybrid assemblies when there is enough ONT data. However, for indels, early interrupted ORFs and recovered BUSCO genes there is a substantial difference between ONT-only and hybrid assemblies that cannot be addressed by increasing the coverage. This new figure nicely reflects these observations, we thank again the reviewers for this suggestion.

Q: Typo: Page 8, line 21. „idoneal" maybe should be „ideal".

R: Thanks for spotting this error. We have now changed the word.

Q: Typo: Page 5, line 21f. „...is available at as a https://gitlab.com/sirarredondo/long_read_selection Snakemake pipeline" should be „ is available as a Snakemake pipeline at https://gitlab.com/sirarredondo/long_read_selection".

R: Thanks for spotting this typo that occurred while editing the final version of the manuscript. We have now changed the sentence.

Powered by Editorial Manager® and ProduXion Manager® from Aries Systems Corporation

Q: Wording: Page 1, Background. „Bacterial whole-genome sequencing based on short-read sequencing data ...". "Sequencing based on sequencing data" is phrased strangely. Maybe „Bacterial whole-genome sequencing based on short-read technologies..." or „Bacterial whole-genome assemblies based on short-read sequencing data...".

R: We appreciate the suggestion, indeed the repetition of the word 'sequencing' was not optimal. We have followed the edit suggested and consider 'Bacterial whole-genome sequencing based on short-read technologies'

Sebastian Alexander Fuchs & Alexander Dilthey
Reviewer #2: GIGA-D-21-00176 "A high-throughput multiplexing and selection strategy to complete bacterial genomes"

The study by Arredondo-Alonso et al. presents a strategy to select candidate bacterial isolates to be further subjected to long read sequencing in order to provide a complementary sequencing in addition to the already performed, short-read based sequencing of the genomes of those isolates.

I found the study well-structured, and interesting to read. My main comments concern the following points:
Q: A) Conceptually, the authors start from a collection of well-sequenced isolates based on short-read sequencing (SRS). They analyse the genomic diversity in the collection, cluster the isolates based on their difference in orthologous gene content, and select the desired number of isolates to be long-read sequenced based on isolates that are the best representative of "clusters" of diversity:
A1) Page 4. The start of the analysis is the matrix of orthologous genes (pangenome). One may argue that the isolates have already been sufficiently sequenced to obtain reliable estimate of gene content. Otherwise the approach does not work. In that case, the advantage of long reads would mostly be to bridge the contigs, but not to discover new genes, etc. In case the SRS data is not of enough quality, the initial comparison of gene presence-absence would not work anymore. Thus, the authors should comment on what is the threshold to consider good or conversely, not suitable, SRS data to be used in their hybrid approach.

R: We thank the reviewer for this excellent comment. Indeed, as mentioned later in their review, the quality of SRS data is crucial to obtain a confident estimate of the gene content. For this reason, we believe that the best metric to indicate the completeness of the genome is the number of dead-ends present in the SRS assembly graph. At the same time, this is influenced by the SRS coverage obtained, in most cases, a low SRS coverage can result in a high number of dead-ends which can be translated as parts of the genome not sequenced/represented in the SRS graph. The number of dead-ends per genome can be retrieved using popular tools such as Bandage. In addition, as mentioned later by the reviewer, these dead-ends need to be completed with ONT reads which can result in an elevated number of SNPs and indels for that particular genomic region.

Based on these observations and supported by Figure S3, we have recommended and discouraged the use of SRS genomes with more than 5 dead-ends per genome (Discussion, page 16) which would indicate that several genomic regions are not present in the SRS genome. On the contrary, if SRS genomes with a low number of dead-ends were included, a reliable estimation of the gene content can be obtained.

Q: Page 3. The authors write "During the hybrid assembly process, only a fraction of the total number of long-reads generated are required to bridge and span the initial short-read assembly graph and thus a low ONT coverage is sufficient to complete a genome." Can the authors also cite some references for this sentence?

R: We thank the reviewer for pointing this out. We have added the following references to support this sentence:
Wick RR, Judd LM, Gorrie CL, Holt KE (2017) Unicycler: Resolving bacterial genome assemblies from short and long sequencing reads. PLOS Computational Biology 13(6):

Powered by Editorial Manager® and ProduXion Manager® from Aries Systems Corporation

e1005595. https://doi.org/10.1371/journal.pcbi.1005595
Nguyen SH, Cao MD, Coin LJM (2021) Real-time resolution of short-read assembly graph using ONT long reads. PLOS Computational Biology 17(1): e1008586. https://doi.org/10.1371/journal.pcbi.1008586

Q: A2) Pages 4-5. Essentially, the authors apply k-means clustering to the isolate coordinates obtained from the t-SNE mapping of the genomic differences. It is thus not surprising that the k-means algorithm performs well (clustering data originating from an Euclidean space would indeed recover most of the variance by k-means clustering, because it also works in that space). The question is whether the k-means clustering could/should not rather be applied to the original (Ds,s) matrix. This would partition the original variation from gene content among isolates into distinct clusters. Otherwise the variance that is described is the variance in t-SNE mapping coordinates.

R: We thank the reviewer for this excellent comment. The main reason why we chose to apply the k-means clustering on the t-SNE mapping coordinates is that the algorithm works best with spherical clusters while it struggles with clusters of distinct shapes which could be present in the original matrix. The t-SNE algorithm ensures that the obtained clusters are spherical and thus are optimal to be searched with the k-means algorithm. We have now clarified in the Methods section 'Long-read selection based on existing short-read assemblies' (page 5) and Results section 'An isolate long-read selection spanning the genome diversity inherent in a short-read collection' (page 10) that the variance described refers to the variance of the t-SNE mapping rather than the variance of the original matrix.

Q: A3) The authors justifiably want to maximize the amount of genomic diversity they can recover from their collection by sequencing. Yet, their approach selects the most common genomic profile among isolates from a given cluster, so they maximize the chance of finding the same genes as mostly found in other isolates from the same k-means cluster. If the strategy truly is to maximize the genomic diversity, would more extreme or divergent profiles in a cluster be more interesting to sequence? Also the isolates with unusual parameters (low coverage, different GC%, a lot of accessory genes, many plasmids…) be more logical candidates to be first sequenced to expand the list of known genes in the collection?

R: This is an outstanding comment. As argued by the reviewer, in our proposed approach we select the 'average' isolate in each k-means cluster by retrieving the isolate with the lowest Euclidean distance to its cluster centroid. Isolates belonging to a particular k-means cluster carrying non-core genes only shared by a minority of the isolates in the cluster will not be selected since they will show a higher Euclidean distance to their centroid compared to other isolates in the cluster. Now, we mention in the Discussion (page 16) the possibility suggested by the reviewer of selecting isolates more distant to its centroid compared to other samples from the cluster with the goal of selecting isolates with a more extreme genome profile.

We believe this is a very important point and we have extended the discussion on this topic (see Discussion, page 16) and argued the pros/cons of the proposed selection approach. The main reason for not selecting first isolates with unusual parameters is that for downstream analyses, we rely on the reference genomes obtained. Thus, the selection of the 'average' isolate in the cluster represents best the gene content for that particular k-means cluster. Following this, the complete genomes resulting from isolates with a low SRS coverage could contain a higher number of SNPs and indels since these absent genomic regions should be completed with ONT reads. However, this could be circumvented by the excellent suggestion of the reviewer of using a threshold to exclude isolates with low coverage.

Furthermore, if the number of centroids is sufficiently high those isolates with unusual parameters will tend to form individual k-means clusters and will be selected whereas if only a few isolates can be selected for ONT sequencing, we argue that choosing those that represent the average gene content may benefit downstream analyses.

However, we strongly agree with the reviewer that other selection approaches may be more suitable depending on the ultimate goal for which the complete genomes are

Powered by Editorial Manager® and ProduXion Manager® from Aries Systems Corporation

required.

Q: B) The authors claim that the majority of the 96 isolates were fully recovered by their approach (Page 3), but this may largely be due to the sequencing data obtained via short read sequencing. Indeed, with 10.71 Gbp (page 10) obtained for the 96 isolates together, this represents about 25x coverage assuming 4.5 MB per E. coli genome (assuming homogeneous coverage for normalized libraries). With this low coverage, the ONT data alone could not be enough to produce complete and high-quality genome sequences. Similarly, if the SRS data was of poor quality (say 10x sequenced), then the ONT data at this obtained coverage would not help much recovering full and high-quality genome sequences. Similarly, the evaluation of the assembly quality based on ONT data alone (Flye) pages 12-13 may not be fair in that context, because of the low sequencing depth imposed by the experimental study design.

R: We thank the reviewer for emphasizing these important points The ONT output (10.71 Gbp) is indeed a problem to obtain high-quality genomes only considering the long-reads obtained. During the evaluation of ONT-only assemblies, we aimed to show that with the imposed experimental setup the accuracy of the genomes with a lower ONT read depth is problematic. Thus, we have now emphasized in the Discussion (page 16) that the Flye results presented are heavily affected by the low ONT data obtained for some of the isolates due to an unequal distribution of ONT reads per barcode in the flow cell and that the assembler performs much better as shown in Figure 5 at isolates with high ONT data. Thus, as we report now in the Discussion, the reported performance of Flye and Medaka is only informative when considering the constraints imposed by our experimental setup.

In the same manner, the evaluation of the SRS data was explored in Figures S2 and Figures S3 to remark the importance of considering high-quality SRS data to obtain a full and high-quality genome. We have now added in the Discussion the importance of including isolates with a low number of dead-ends and indicated as 5 the optimal threshold to consider isolates for the long-read selection.

Q: C) The authors should indicate what was their expected coverage per isolate (page 6). Multiplexing 96 genomes on one flow cell would necessarily provide low coverage for many cases. Also Page 6, the authors seem not to have normalized the concentration of each library prior to sequencing to allow for more homogeneous representation of each isolate in the library.

R: Thanks for remarking this. We have now indicated in the Results section 'Uneven distribution of ONT reads in the 96 multiplexing approach' (page 11) the expected genome coverage considering a genome size of 5Mbp (chromosome + extrachromosomal elements).

We have now indicated in the Methods section 'ONT library preparation' (page 7) that prior to library preparation, the samples were adjusted to 400 ng to normalize the concentration of the samples in the library.

Q: D) Page 7 the authors wrote "a quality phred score of 20 (--mean_q_weight 20), retaining 90% of the total number of ONT reads (--keep_percent 90) from a maximum 40x
coverage (--target_bases)." I doubt that any ONT read is left with such a hight Phred score! Please revise if necessary.

R: We thank the reviewer for spotting this error. Indeed, this flag from Filtlong https://github.com/rrwick/Filtlong does not correspond to the Phred score but a weight given by Filtlong to obtain a mean quality score. We have now changed this in the Methods section 'Hybrid assemblies' (page 7) with the explanation of the flag given by Filtlong.

Q: E) Plasmid presence. The authors did not indicate whether the same gDNA was used for both SRS and ONT sequencing. This may be of importance to judge whether

a plasmid is present or not given a specific sequencing technology and assembly approach are used. The isolate may have lost its plasmid(s) due to culturing conditions etc. so the comparison may be biased.

R: This is an excellent point. We used different gDNA for the SRS and ONT sequencing due to the fact that the short-read collection was sequenced in a previous study (Gladstone et al. 2021, https://doi.org/10.1016/S2666-5247(21)00031-8). For this reason, we had to renew the growth of isolates and perform another DNA extraction step. As mentioned by the reviewer, this can introduce some bias when comparing extrachromosomal elements between ONT and Illumina libraries. We have now explicitly stated this limitation in the Discussion (page 17).

Q: F) Page 15 (Discussion). The authors wrote "In the hybrid assemblies, the accuracy of the complete genomes is unaffected by the ONT read depth since, in general, the long-reads are only used as bridges to unequivocally connect short-read contigs". This all depends on how good the assemblies based on SRS data are. If many genomic regions are missing after SRS, the ONT data would be the only reads available to cover those missing regions, hence the error rate of ONT will apply to those regions.

R: Thanks for this excellent point. In the same paragraph, we mention that the assembly quality is dependent on the initial quality of the short-read graph. And also state, as mentioned by the reviewer, that the regions missing in the SRS (dead-ends) in the final sequence would rely on the ONT reads since there are no short-reads available.

Q: Spelling mistakes, suggested edits:
- Abstract. The sentence "we propose a long-read isolate selection strategy that optimizes a representative selection of isolates" is not clear. I don't recommend aggregating succinctly the terms in "long-read isolate selection strategy", as this leads to conceptual unclarity. The same is true at several other places in the manuscript, e.g. Page 3 "Long-read selection based on existing short-read assemblies", "From a large collection of short-read isolates". Page 5 "To showcase the proposed long-read selection"… Please check throughout the manuscript.

R: This is an excellent point. We have rephrased in the whole manuscript the combination of words ‘long-read selection’ for ‘isolate selection’ to improve the clarity of the text.

Q: Page 2: Add a reference to "These long-reads can typically span repeat elements in a bacterial genome producing a contiguous assembly consisting of single and circular contigs per replicon
(chromosome and/or plasmids)."

R: Thanks for this remark. We have added the following references:

Loman, N., Quick, J. & Simpson, J. A complete bacterial genome assembled de novo using only nanopore sequencing data. Nat Methods 12, 733–735 (2015). https://doi.org/10.1038/nmeth.3444
Judith Risse, Marian Thomson, Sheila Patrick, Garry Blakely, Georgios Koutsovoulos, Mark Blaxter, Mick Watson, A single chromosome assembly of Bacteroides fragilis strain BE1 from Illumina and MinION nanopore sequencing data, GigaScience, Volume 4, Issue 1, December 2015, s13742–015–0101–6, https://doi.org/10.1186/s13742-015-0101-6

Q: Page 2: Last sentence. The authors do not mention existing software that address the basecalling issues in ONT data (e.g. nanopolish, medaka) and significantly enhance the quality of the consensus sequences, based on ONT data alone.

R: Excellent point. We have added a sentence in this paragraph to explain that an improved consensus sequence can be generated using these two popular tools (Nanopolish and Medaka).

Q: Page 7: Please rephrase "To map the Illumina reads against each of the

	nucleotides assembled by Unicycler".<br><br>R: Thanks for this remark. We have rephrased this sentence.<br><br>Q: Page 8: "idoneal"?<br><br>R: Thanks for spotting this error. We have now used the word 'ideal'.
Additional Information:	
Question	Response
Are you submitting this manuscript to a special series or article collection?	No
**Experimental design and statistics**<br><br>Full details of the experimental design and statistical methods used should be given in the Methods section, as detailed in our Minimum Standards Reporting Checklist. Information essential to interpreting the data presented should be made available in the figure legends.<br><br>Have you included all the information requested in your manuscript?	Yes
**Resources**<br><br>A description of all resources used, including antibodies, cell lines, animals and software tools, with enough information to allow them to be uniquely identified, should be included in the Methods section. Authors are strongly encouraged to cite Research Resource Identifiers (RRIDs) for antibodies, model organisms and tools, where possible.<br><br>Have you included the information requested as detailed in our Minimum Standards Reporting Checklist?	Yes
**Availability of data and materials**<br><br>All datasets and code on which the conclusions of the paper rely must be either included in your submission or deposited in publicly available repositories	Yes

(where available and ethically appropriate), referencing such data using a unique identifier in the references and in the “Availability of Data and Materials” section of your manuscript.<br><br>Have you have met the above requirement as detailed in our Minimum Standards Reporting Checklist?	

Main Manuscript - Clean Version

Click here to access/download;Manuscript;MM_Revision_Clean_Version_Su

A high-throughput multiplexing and selection strategy to complete bacterial genomes

Authors

Sergio Arredondo-Alonso[1,2*], Anna K. Pöntinen[1], François Cléon[3], Rebecca A. Gladstone[1], Anita C. Schürch[4], Pål J. Johnsen[3], Ørjan Samuelsen[3,5], Jukka Corander[1,2,6]

1. Department of Biostatistics, University of Oslo, Oslo, Norway
2. Parasites and Microbes, Wellcome Sanger Institute, Cambridge, UK
3. Department of Pharmacy, Faculty of Health Sciences, UiT The Arctic University of Norway, Tromsø, Norway
4. Department of Medical Microbiology, UMC Utrecht, Utrecht, the Netherlands
5. Norwegian National Advisory Unit on Detection of Antimicrobial Resistance, Department of Microbiology and Infection Control, University Hospital of North Norway, Tromsø, Norway
6. Department of Mathematics and Statistics, Helsinki Institute of Information Technology (HIIT), FI-00014 University of Helsinki, Helsinki, Finland

* Corresponding author

Abstract

Background: Bacterial whole-genome sequencing based on short-read technologies often results in a draft assembly formed by contiguous sequences. The introduction of long-read sequencing technologies permits to unambiguously bridge those contiguous sequences into complete genomes. However, the elevated costs associated with long-read sequencing frequently limit the number of bacterial isolates that can be long-read sequenced.

Here we evaluated the recently released 96 barcoding kit from Oxford Nanopore Technologies (ONT) to generate complete genomes on a high-throughput basis. In addition, we propose an isolate selection strategy that optimizes a representative selection of isolates for long-read sequencing considering as input large-scale bacterial collections.

Results: Despite an uneven distribution of long-reads per barcode, near-complete chromosomal sequences (assembly contiguity = 0.89) were generated for 96 *Escherichia coli* isolates with associated short-read sequencing data. The assembly contiguity of the plasmid replicons was even

higher (0.98) which indicated the suitability of the multiplexing strategy for studies focused on resolving plasmid sequences. We benchmarked hybrid and ONT-only assemblies and showed that the combination of ONT sequencing data with short-read sequencing data is still highly desirable: (i) to perform an unbiased selection of isolates for long-read sequencing, (ii) to achieve an optimal genome accuracy and completeness, and (iii) to include small plasmids underrepresented in the ONT library.

Conclusions: The proposed long-read isolate selection ensures the completion of bacterial genomes that span the genome diversity inherent in large collections of bacterial isolates. We show the potential of using this multiplexing approach to close bacterial genomes on a high-throughput basis.

Introduction

Whole-genome sequencing (WGS) of bacterial isolates has dramatically increased its routine presence in clinical genomics and epidemiological investigations [1]. The possibility of using short-read technologies, affordable and generally accurate in terms of their sequencing reads, has permitted tracking the presence of particular sequencing types, assessing the presence of single-point mutations, or identifying antimicrobial resistance (AMR) genes in collections of thousands of bacterial isolates [2–4]. These applications are fundamental during outbreak detections and investigations, for which WGS has overcome some limitations associated with classical epidemiological techniques [2,5,6]. However, the read length associated with these short-read technologies (from 150 bp to 300 bp) cannot unambiguously span the presence of repeat elements such as insertion sequences (IS). This results in a fragmented assembly, typically formed by contiguous sequences (contigs) of unknown order. In particular, determining the genome context (chromosome, plasmid, phage) of genes typically surrounded by IS elements (e.g AMR genes) is challenging in draft assemblies [7,8].

Long-read sequencing technologies such as Oxford Nanopore Technologies (ONT) can generate genomic libraries with an average read length between 10-30 kbp [9], but with a lower associated raw read accuracy of ~97% (phred score ~15) depending on the pore chemistry [10]. These long-reads can typically span repeat elements in a bacterial genome producing a contiguous assembly consisting of single and circular contigs per replicon (chromosome and/or plasmids) [11,12]. However, the sequence accuracy of these complete genomes can suffer from incorrect basecalling of short homopolymer sequences resulting in early termination of open reading frames (ORF) in protein-coding genes [13]. This limitation can be mitigated by creating an improved consensus sequence and variant calls, reusing the ONT sequencing data with tools such as Nanopolish [11] or Medaka [14].

An attractive alternative is to combine short- and long-read technologies to generate complete and accurate genomes in a process called hybrid assembly [15]. A frequent scenario encountered by researchers is that large collections of bacterial isolates have been massively short-read sequenced and only a subset of these isolates can be further selected for long-read sequencing. Therefore, selecting isolates for long-read sequencing is a non-trivial and crucial step that can affect subsequent analyses based on the resulting complete genomes.

There are different strategies to decrease the price of completing a genome with ONT sequencing [16,17]. Recently, Lipworth et al. [18] showed that usage of wash-kits coupled with shorter sequencing times can be optimized to obtain 36 complete genomes per single flow cell, achieving a reduction in long-read sequencing costs of 27%. During the hybrid assembly process, only a fraction of the total number of long-reads generated are required to bridge and span the initial short-read assembly graph and thus a low ONT coverage is sufficient to complete a genome [19,20].

The possibility of increasing the number of multiplexed isolates per flow cell is an alternative to the usage of wash-kits that reduces hands-on-time and avoids contamination issues between libraries. Recently, ONT has released a new barcoding kit allowing multiplexing 96 isolates in the same sequencing library.

Here, we evaluated the degree of completeness and accuracy in bacterial genomes generated using the new ONT barcoding kit for 96 bacterial isolates. In addition, we provide a step-by-step computational workflow to perform an unbiased selection of isolates for long-read sequencing based on the presence/absence of genes. as previously applied in two large bacterial collections of isolates [21,22].

The approach described in this study generated near-complete genomes for the majority of 96 *E. coli* isolates. The computational workflow proposed can be used to maximize and complete a representative selection of isolates from large-scale bacterial collections.

Methods

Isolate selection based on existing short-read assemblies

From a large collection of short-read isolates, frequently only a subset of isolates can be completed with long-read sequencing. We propose the following approach to select a representative subset of isolates for a population sample:

1. Define *M* as the presence/absence matrix of orthologous genes created by pangenome tools such as Roary [23] or Panaroo [24]. *M* is a binary matrix with *s* x *g* dimensions, in which *s* is the total number of isolates (samples) present in the collection and *g* corresponds to the total number of orthologous genes predicted.

$$M_{s,g} = \begin{pmatrix} m_{1,1} & m_{1,2} & \cdots & m_{1,g} \\ m_{2,1} & m_{2,2} & \cdots & m_{2,g} \\ \vdots & \vdots & \ddots & \vdots \\ m_{s,1} & m_{s,2} & \cdots & m_{s,g} \end{pmatrix}$$

2. We transform *M* into a Jaccard distance matrix *D* with *s* x *s* dimensions using the R function *parDist* (method = 'fJaccard ') provided in the R package parallelDist (version 0.2.4) https://github.com/alexeckert/parallelDist.

$$D_{s,s} = \begin{pmatrix} d_{1,1} & d_{1,2} & \cdots & d_{1,s} \\ d_{2,1} & d_{2,2} & \cdots & d_{2,s} \\ \vdots & \vdots & \ddots & \vdots \\ d_{s,1} & d_{s,2} & \cdots & d_{s,s} \end{pmatrix}$$

   For instance, the element $d_{1,2}$ can be defined as the similarity distance between the genes predicted for the first isolate and second isolate:

$$M_1* = \{m_{1,1}, m_{1,2}, ..., m_{1,g}\}$$
$$M_2* = \{m_{2,1}, m_{2,2}, ..., m_{2,g}\}$$
$$d_{1,2} = \frac{|M_{1*} \cap M_{2*}|}{|M_{1*} \cup M_{2*}|}$$

3. Next, the distance matrix *D* can be dimensionally reduced using the t-distributed stochastic neighbor embedding algorithm (t-sne) [25] which results in a new matrix *T* with only two dimensions. In this step, we use the R package Rtsne (version 0.15) https://github.com/jkrijthe/Rtsne considering as default a perplexity value of 30.

4. Next, we use the k-means algorithm [26] with *T* as an input data and define a number of centroids *c* such that it corresponds to the desired number of isolates to be long-read sequenced. A random initialization of the centroids permits defining the clusters *k* in *T* by assigning each point t_i to its closest centroid. For each cluster C_k, the algorithm updates the

position of the centroid by computing the average Euclidean distance of each point t_i against the centroid. The within-square variation V for a particular cluster C_k can be defined as:

$$V(C_k) = \sum_{t_i \in C_k} (t_i - \mu_k)^2$$

This process is repeated until convergence is reached, and thus the position of the centroids c in T no longer varies or the maximum number of iterations is reached (default = 1,000 iterations).

To run the algorithm, we use the function *kmeans* provided in the R package stats (version 3.6.3). For a fixed number of centroids c, we run the k-means algorithm using 10,000 distinct initializations and select the initialization with the highest ratio between-cluster sum of squares and the total sum of squares (between_ss/tot_ss).

$$tot.within\_ss = \sum_{k=1}^{k} V(C_k)$$

$$tot\_ss = \frac{1}{N} \sum_{i,i' \epsilon \{1,2,\ldots N\}} (t_i - t_{i'})^2$$

$$between\_ss = tot\_ss - tot.within\_ss$$

N corresponds to the total number of points t_i present in T.

The ratio (between_ss/tot_ss) is considered as the percent of total variance from T explained by the chosen number of centroids (clusters). The relationship between the percent of total variance explained and the number of centroids can be considered to visually determine the ideal numbers of clusters required to capture the diversity present in the collection (Elbow method).

5. For each cluster C_k, we select for long-read sequencing the isolate t_i with the lowest Euclidean distance with respect to its associated centroid c.

$$long\, read\, t_i = \min_{t_i \in C_k} t_i - c$$

6. The tsne matrix T together with the final coordinates of the centroids c and the t_i isolates selected for long-read sequencing are plotted using the R package ggplot2 (version 3.3.3).

The proposed workflow aims at capturing as comprehensive representation of the gene content variation across the lineages in the target population as possible and is available at as a https://gitlab.com/sirarredondo/long_read_selection Snakemake pipeline [27] that only requires (i) a presence/absence matrix in the same format as created by Roary/Panaroo and (ii) the desired number of long-read isolates.

Collection of Illumina short-read assemblies

To showcase the proposed long-read selection, we considered the Norwegian *Escherichia coli* collection of 3254 isolates causing bloodstream infections recently described by Gladstone et al. [28]. The DNA extraction of this collection was performed using the DNeasy 96 Blood and Tissue kit (QIAGEN, Hilden, Germany), the isolates were short-read sequenced with the Illumina HiSeq platform and short-read contigs were created using VelvetOptimiser (version 2.2.5) https://bioinformatics.net.au/software.velvetoptimiser.shtml and Velvet (version 1.2.10) [29]. Furthermore, an assembly improvement step was applied to the assembly with the best N50 and contigs were scaffolded using SSPACE (version 2.0) [30] and sequence gaps filled using GapFiller (version 1.11) [31]. These short-read contigs will be later considered to compare the accuracy of the hybrid and ONT-only assemblies (section Genome accuracy and completeness).

The presence/absence of orthologous genes defined by Panaroo (version 1.0.2) [24] and PopPUNK lineages (version 2.0.2) [32] associated with the isolates were also extracted from Gladstone et al. [28] and considered as input for the long-read selection process. As an example, we fixed the number of centroids to 96 and considered the between_SS/tot_SS ratio to estimate if the maximum number of isolates that can be multiplexed in an ONT sequencer would suffice to capture the genomic diversity of this particular collection.

We used the selection procedure described above on the *E. coli* collection of 3254 isolates for the following two purposes: i) to showcase the percent of variance (ratio between_ss/tot_ss) recovered by the pipeline with an arbitrary number of isolates (n = 96), and ii) to select a large number of isolates (1085) that are planned to be sequenced with ONT in future.

Out of these 1085 selected isolates, we explored the recently released ONT SQK-NBD110-96 barcoding kit to sequence 96 of these bacterial isolates. This run was crucial to define whether the rest of the isolates (n = 989) could be completed using this multiplexing kit or whether a different strategy would be preferable.

DNA isolation for ONT libraries

In total, 96 *E. coli* isolates, originally from clinical samples of human bloodstream infections, were grown on MacConkey agar No 3 (Oxoid Ltd., Thermo Fisher Scientific Inc., Waltham, MA, US) at 37°C, and individual colonies were picked for overnight growth in LB (Miller) broth (BD, Franklin Lakes, NJ, US) at 37°C in 700 rpm shaking. High-molecular-weight (HMW) genomic DNA from cell pellets of 1.6 ml overnight cultures was extracted using MagAttract® HMW DNA Kit (Qiagen, Hilden, Germany) according to the manufacturer's instructions and using a final elution volume of 100 µl. DNA concentration and integrity were verified using NanoDrop One spectrophotometer (Thermo Scientific) and the Qubit dsDNA HS assay kit (Thermo Fisher Scientific) on a CLARIOstar microplate reader (BMG Labtech, Ortenberg, Germany).

ONT library preparation

Prior to library preparation, the samples were adjusted to 400 ng. The ONT library was prepared using SQK-NBD110-96 barcoding kit, and 40 fmol was loaded onto flow cells.. Sequencing was run for 72 hours on GridION using FLO-MIN106 flow cells and MinKNOW v.20.10.6 software. Basecalling was conducted with the high-accuracy basecalling model and demultiplexing was performed by using Guppy v.4.2.3.

Hybrid assemblies

Porechop (version 0.2.4) https://github.com/rrwick/Porechop was used to trim and remove ONT adapters with default parameters. Filtlong (version 0.2.0) https://github.com/rrwick/Filtlong was used to filter the ONT reads considering a minimum length of 1 kbp (--min_length 1000), a weight given to the mean quality score of 20 (--mean_q_weight 20), retaining 90% of the total number of ONT reads (--keep_percent 90) from a maximum 40x coverage (--target_bases). Unicycler (version 0.4.7) [19] was run using the normal mode to perform a hybrid assembly with the Illumina trimmed reads and ONT reads retained after Filtlong.

We extracted the number of segments (contigs), links (edges), N50 and size of the components present at the resulting hybrid assembly graph. For each component, we defined its contiguity as:

$$Contiguity = \frac{N50\ Component}{Component\ Size}$$

Mlplasmids (version 1.0.0) [33] was used with the *E. coli* model to confirm the origin (plasmid- or chromosome-derived) from the longest segment of each component.

lllumina and ONT depth per replicon

Illumina reads were mapped against the genomeassembled by Unicycler, using Bowtie2 (version 2.4.2) [34] with the argument --very-sensitive-local, a minimum and maximum fragment length of 0 (-I 0) and 2000 respectively (-X 2000). The ONT reads were mapped against Unicycler assemblies using bwa mem (version 0.7.17) [35] and indicating the ready type (-x) as ont2d. Samtools (version 1.12) [36] was used with the commands sort, index and depth to process the alignments and retrieve the number of reads covering an individual nucleotide.

For each component present in the graph file given by Unicycler, we considered its longest contig and computed the average number of reads. To normalize the contig depth with respect to its chromosome, we divided the average depth of the contig against the average depth of the longest contig assembled by Unicycler (longest chromosome segment).

ONT-only assemblies and long-read polishing

Flye (version 2.8.3-b1695) [37] was run with all the QC-passed ONT reads (phred score > 7) available per barcode (--nano-raw), specifying the option to recover plasmids (--plasmids), indicating an expected genome size of 5 Mbp (--genome-size 5m) and considering 3 polishing rounds (--iterations 3). For each component present in the assembly graph, we extracted the number of segments (contigs), links (edges), N50, component size and defined its component contiguity. Mlplasmids (version 1.0.0) [33] was used with the *E. coli* model to confirm the origin (plasmid- or chromosome-derived) from the longest segment of each component.

Following the recommendations to further polish Flye assemblies https://github.com/rrwick/Trycycler/wiki/Guide-to-bacterial-genome-assembly#13-medaka, we used Medaka (version 1.2.5) https://github.com/nanoporetech/medaka with the model 'r941_min_high_g360' and considered as input all the passed ONT reads available per barcode. In addition, we performed a consecutive round of Medaka to observe whether additional rounds of polishing could improve the resulting genomes.

Genome accuracy and completeness

Quast (version 5.0.2) [38] was used to compute alignments with a minimum size of 5 kbp (--min-alignment 5000) considering as reference short-read contigs assembled with Velvet (section Collection of Illumina short-read assemblies) from the same isolate against Unicycler, Flye and Medaka-polished contigs. For each reference contig, we kept the best alignment (--ambiguity-usage one). The average number of single-nucleotide polymorphisms (SNPs) and insertions and deletions

(indels) per 100 kbp were considered as a reference-based metric of the genome accuracy shown by the hybrid and ONT-only assemblies.

For Unicycler assemblies, we also extracted the contigs present in the file ‘001_best_spades_graph.gfa’ corresponding to an optimal SPAdes assembly graph. Unicycler uses a range of k-mer sizes and computes a score between the resulting number of contigs and dead-ends to choose the SPAdes graph idealfor the long-read bridge process. These SPAdes contigs were also compared using Quast against the reference contigs assembled by Velvet. This was relevant to determine the SNPs and indels per 100 kbp present at the SPAdes contigs. In this manner, we could assess if the accuracy (SNPs, indels) estimated for the hybrid assemblies was influenced by: (i) differences between the short-read assemblers, and/or (ii) regions of the genome from which its sequence is determined by the ONT reads, such as regions connecting dead-ends in the SPAdes graph.

The ideel test https://github.com/mw55309/ideel was used to obtain the number of early terminated ORFs in the assemblies [13]. Diamond (version 2.0.8) [39] was run with the blastp algorithm [40], specifying only a single target sequence per alignment (--max-target-seqs 1), a block size of 12 (-b12) and index chunk of 1 (-c1) against an index of the UniProt TREMBL database (retrieved in April 2021). For each hit, the ratio between query length and its own length was considered to assess the presence of interrupted ORFs (ratio < 0.9). This assesses whether the length of the predicted proteins is shorter than their closest hit, most likely caused by the introduction of a stop codon. Importantly, the total number of interrupted ORFs may include true pseudogenes, however, most of these hits are considered as non-true errors introduced due to a low sequencing accuracy. This ideel test provided a reference-free approach to assess the number of indels present in the final assemblies.

BUSCO (version 5.1.2) [41] with the genome mode (-m genome) and the lineage dataset (-l) ‘enterobacterales_odb10’ was used to assess the presence of 440 single-copy orthologous genes. BUSCO uses the following notation to annotate the genes as: complete (length within two standard deviations of the group mean length), fragmented (partially recovered) and missing (totally absent). The number of BUSCO complete genes was considered as a metric to assess the completeness of each assembly.

Results

An isolate selection spanning the genome diversity inherent in a short-read collection

In large bacterial collections for which possibly thousands of isolates are short-read sequenced, the selection of a subset of isolates for long-read sequencing is crucial to obtain a representative set of complete genomes spanning the genomic diversity present in the collection.

We propose the following approach summarised in the following steps (see Methods, for a formal description): i) consider the presence/absence matrix of orthologous genes computed by established pangenome tools such as Roary or Panaroo, ii) compute a distance matrix based on Jaccard distances, isolates are compared based on their shared number of orthologous genes, iii) reduce the dimensionality of the distance matrix using tsne by preserving local structure, iv) cluster the tsne dimensionally reduced matrix with the k-means algorithm, indicating as the number of centroids the desired number of long-read isolates and v) select the isolate with the closest Euclidean distance to its centroid. The proposed approach is fully available as a Snakemake pipeline at https://gitlab.com/sirarredondo/long_read_selection

We showcase the approach on a set of 3254 short-read sequenced isolates from a Norwegian longitudinal population genomic study of *E. coli* causing bacteraemia [28]. The number of isolates selected for long-read sequencing can be specified by fixing the number of centroids in the pipeline. For example, in this collection (Figure 1), we estimated that selecting 96 long-read isolates would capture ~ 99.9% of the total variance present in the tsne matrix computed from Jaccard distances. The selection of the 96 isolates indicated in Figure 1 would capture all orthologous genes with a frequency higher than 0.05 (Figure S1). As expected, a substantial proportion of genes with a frequency of around 0.01 are not present in these 96 isolates (Figure S1) since those genes are rarely shared by other isolates from the same k-means cluster.

From the same collection of 3254 isolates, we performed an independent selection of 1085 *E. coli* isolates for long-read sequencing using the proposed selection strategy. These isolates also belong to the Norwegian collection described above [23] for which short-read sequencing data are publicly available.

We explored the possibility of completing these 1085 genomes by using the recently released ONT native barcode expansion kit. This kit allowed multiplexing in the same MinION flowcell up to 96 isolates and potentially recovering complete genomes in a high-throughput manner.

In the following sections, we evaluated the complete genomes obtained by multiplexing 96 of these 1085 isolates, in the same MinION flowcell. These 96 isolates differ from those shown in Figure 1, since they were selected considering a distinct number of centroids.

Uneven distribution of ONT reads in the 96 multiplexing approach

We used the recently released barcoding kit from ONT (SQK-NBD110-96) to multiplex 96 *E. coli* isolates (96 out of 1085 isolates) in the same MinION flow cell. The ONT sequencing run generated a total of 10.71 Gbp QC-passed basecalled reads (average phred score > 7) that could be confidently assigned to a barcode, with an average N50 read length of 20.98 kbp (Figure 2). Considering a genome size of 5Mbp, the expected coverage was around ~22x per isolate. From the passed reads, the average ONT phred score corresponded to 12.48 equivalent to a read accuracy of 94.35 %. As previously reported for other multiplexing kits [16], we observed an uneven distribution of reads available per barcode which resulted in a large variation in the number of bases available per barcode (mean = 111.53 Mbp, median = 103.45 Mbp). This difference ranged from 3.37 Mbp (coverage ~ 0.67x) for barcode 68 to 244.00 Mbp (coverage ~ 48x) in barcode 73. Prior to the hybrid assemblies, ONT reads were filtered using Filtlong based on quality and length (see Methods), slightly reducing the number of reads available (mean = 94.36 Mbp, median = 85.39 Mbp). A complete description of ONT statistics per barcode is given in Suppl. Table S1.

Evaluation of the hybrid assemblies

First, we focused on the chromosome sequence by analysing the largest component present in the hybrid assembly graph. In the 96 samples, the chromosome was present in a component with an average size of 5.0 Mbp (median = 5.03) and 11.39 contigs (median = 2.0) respectively (Figure 3). Despite the average number of contigs forming the chromosome, the contiguity (N50/component size) of the chromosome replicon was 0.89 (median = 1.0) which indicated that the chromosome component was assembled, for most samples, in a large contig (Figure 3). Furthermore, in 48 samples (50%) the chromosome resulted in a single circular contig (contiguity = 1.0) (Suppl. Table S2).

We observed a positive correlation (pearson corr. = 0.41) between the chromosome contiguity and the number of ONT reads available during the hybrid assembly (Figure 3). We assessed that an approximate ONT depth of ~5x was minimally required to achieve a perfect chromosome assembly (contiguity = 1.0). The lowest contiguity values corresponded to two isolates with a poor ONT coverage (< 1x) (barcode 65 and barcode 68). Overall, the isolates with an inferior contiguity value (threshold < 0.9) (18 barcodes, ~19%) had an associated low number of ONT reads available during the hybrid assembly (mean = 42.64 Mbp, coverage ~ 8.53x).

Next, we assessed the rest of the components present in the hybrid assembly graphs. The largest segment of each component was predicted with mlplasmids to confirm its plasmid origin. For medium and large plasmid components (size > 10 kbp) (n = 132), we obtained an average contiguity of 0.98 (median 1.0) and single circular contigs were retrieved for 118 components (89.4%) (Table 1). On

average, the 132 plasmid components were formed by 1.79 contigs (median = 1.0). For the isolates with a low chromosome contiguity (< 0.9), the average plasmid contiguity was still 0.97 (median = 1.0) which indicated the suitability of the pipeline for plasmid reconstruction purposes. The correlation between plasmid contiguity and ONT reads available was weaker (pearson corr. = 0.16) than for the chromosomal component. Even for isolates with a poor ONT coverage, the contiguity values were close to 1.0 indicating that only a few long reads are sufficient to resolve plasmid components. The small plasmids (size < 10 kbp, n = 78) had an average contiguity of 0.99 (median = 1.0) and single circular contigs were retrieved for 74 plasmids (94.9%). The absence of repeat sequences in small plasmids permits to already obtain circular replicons by only using short-read sequencing assemblies and thus ONT reads are not required.

Underrepresentation of small and medium plasmids in the ONT library

For each component present in the Unicycler assemblies, we determined its Illumina-read and ONT-read relative depth considering the average chromosomal depth as basis for the normalization, as previously performed [16]. This analysis was fundamental to confirm whether small plasmids were underrepresented in the ONT library preparation, as recently reported for ONT ligation kits [42].

As shown in Figure 4, small plasmids (size < 10 kbp) were strongly underrepresented in the ONT read output (log2 -0.63) in contrast to the Illumina read output (log2 4.79). For 7 small plasmids, we did not observe any ONT reads covering these replicons. These plasmids are usually present in high copy numbers in the cell as a survival and inheritance mechanism [43]. Therefore, we expected that these replicons would be overrepresented in the sequencing output resembling the plasmid read output given by Illumina (Figure 4). For medium plasmids (from 10 to 50 kbp), we also observed the same trend even though the underrepresentation in the ONT library was less pronounced (log2 -0.40). Notably, we observed the opposite trend for large plasmids (> 50 kbp) for which there was an underrepresentation in the Illumina library (log2 -0.60) compared to the ONT library (log2 0.18).

ONT-only assemblies

To evaluate whether Illumina reads are still required to obtain accurate genomes, we used Flye and Medaka to perform and polish assemblies based only on ONT reads. The largest component in the assembly graph had an average size of 4.45 Mbp (median = 4.98 Mbp) and 1.16 contigs (median = 1.0) (Suppl. Table S3). Flye failed to produce an assembly for 2 isolates (barcode 65 and barcode 68) for which the number of ONT reads generated was below 5 Mbp (Suppl. Table S1). In 71 samples

(76%), the chromosome was represented by a single circular contig. The average chromosomal contiguity was 0.98 (median = 1.0) which indicated a higher contiguity with respect to the hybrid assemblies. However, the average size of the largest component (mean = 4.45 Mbp, median = 4.98 Mbp) was shorter than for Unicycler assemblies (mean = 5.0 Mbp, median = 5.03 Mbp). This indicated that for some isolates the size of the largest component did not match with the expected replicon size (~ 5Mbp).

The analysis of the rest of the components present in Flye assemblies (n = 346) revealed a high number of replicons with a chromosome origin (n = 193) indicating fragmentation of the chromosome into several components. For the components with a plasmid origin (n = 153), we obtained single circular contigs for 120 plasmids (78.43%). We observed a clear difference between the hybrid and ONT-only assemblies with respect to small plasmids (size < 10 kbp). Only 12 small circular plasmids were recovered in the Flye assemblies, in comparison to the 78 small plasmids present in the hybrid assembly. The absence of these plasmids in the Flye assemblies could be explained by their underrepresentation or complete absence in the total ONT read output, as shown in the section above.

Genome accuracy and completeness of hybrid and ONT-only assemblies

To compare the accuracy of the resulting genomes, the hybrid assemblies and Flye assemblies were compared in terms of SNPs and indels considering both reference-based and reference-free methodologies (see Methods). Despite the fact that Flye incorporates a consensus-error module to correct the resulting genome sequences, we polished the Flye assemblies using Medaka and compared the genome accuracy against hybrid (Unicycler) and stand-alone Flye assemblies. In addition, we evaluated whether a second round of polishing with Medaka could improve the accuracy of the genomes.

The hybrid assemblies showed the best accuracy stats with an average of 6.93 SNPs/100 kbp (median = 5.24) and 0.31 indels/100 kbp (median = 0.21), considering as ground truth non-repetitive alignments (> 5 kbp) against short-read contigs generated with Velvet. The SPAdes assemblies created by Unicycler with only short-reads showed an average of 0.85 SNPs/100 kbp (median = 0.28) and 0.06 indels/100 kbp (median = 0.04). This indicated that the accuracy of the hybrid assemblies was affected by the incorporation of error-prone ONT reads into the final genome sequence. The existence of dead-ends in the initial SPAdes graph indicated that parts of the genome were not sequenced with short-reads, thus their sequence had to be completed with long-reads and could not be polished with Illumina reads. As shown in Figure S2, two barcodes (69 and 72) had an elevated number of dead-ends (38 and 43) which resulted in a high number of SNPs/100 kbp (barcode 69: 23.89; barcode 72: 33.36) and indels/100 kbp (barcode 69: 1.25; barcode 72: 1.32).

Flye assemblies exhibited a higher number of errors with an average of 130.17 SNPs/100 kbp (median = 14.68) and 140.82 indels/100 kbp (median = 41.52). We observed a negative correlation between the number of ONT bases generated and the resulting number of SNPs (pearson corr. = -0.27) and indels (pearson corr. = -0.61) (Figures 5A and 5B). The correction performed by Medaka on Flye assemblies showed a reduction in the number of indels (mean = 126.35, median = 22.84) but the number of mismatches remained similar (mean = 130.29, median = 12.57). A second round of polishing Medaka did not significantly improve the number of indels (mean = 124.16, median = 22.37) or mismatches (mean = 130.66, median = 12.57). Again, we observed a negative correlation between the ONT depth and the resulting number of SNPs (pearson corr. = -0.27) and indels (pearson corr. = -0.61) (Figures 5A and 5B). For instance, for barcode 73 with the highest ONT read depth, the ONT-only assemblies had a number of SNPs (Flye = 6.52 SNPs/100 kbp, Medaka = 6.25 SNPs/100 kbp) comparable to Unicycler results (5.58 SNPs/100 kbp) which indicated that an increase in the ONT read depth can be highly beneficial to correct SNP errors. However, in the case of indels, we still observed 26.47 indels/100 kbp and 6.46 indels/100kbp for Flye and Medaka assemblies respectively, which is far from Unicycler results (0.04 indels/100 kbp).

Following on this, we considered a reference-free approach to evaluate the impact of non-corrected indels in the interruption of ORFs using the ideel test [13]. In the hybrid assemblies, the number of promptly terminated ORFs corresponded to 25.07 (median = 21.0) which may include true pseudogenes. For Flye assemblies, on average the number of interrupted ORFs was 1380.64 (median = 772.5) and the correction with Medaka improved the genome accuracy by reducing the number to 1127.99 interrupted ORFs (median = 470.0). An additional round of polishing with Medaka resulted in a similar number of interrupted ORFs (mean = 1103.88, median = 454.5). We observed a negative correlation between the number of ONT bases available and the number of interrupted ORFs present in Flye assemblies (pearson corr. = -0.76) and Medaka polished assemblies (pearson corr. = -0.78) (Figure 4C). We observed a plateau, around ~500 and ~150 ORFs for Flye and Medaka polished assemblies, for which an increase in ONT depth did not translate into a lower number of early terminated ORFs. Even for the isolate with the highest ONT depth (barcode 73, ONT bases 244 Mbp), the number of interrupted ORFs was still 146 for the Medaka polished assembly in comparison to 23 early terminated ORFs for the hybrid assembly.

Lastly, we evaluated the completeness of the genomes searching for complete orthologous genes against a curated set of 440 *Enterobacterales* single-copy conserved genes (BUSCO genes). For the hybrid assemblies, we recovered all BUSCO genes (100%) in contrast to only 76.6% for Flye assemblies. Furthermore, the percentage of missing genes was 10.3% which indicated that the *E. coli* genomes assembled by Flye were not complete. Medaka correction increased the number of recovered

BUSCO genes (mean = 80.5%) and slightly decreased the number of missing genes (mean = 9.3%). A second round of polishing with Medaka did not significantly recover more BUSCO genes (mean = 80.81) or decrease the number of absent genes (mean = 9.15%). For Flye and Medaka assemblies, a strong positive correlation of 0.78 was observed for both strategies between the ONT depth and the number of BUSCO complete genes (Figure 4D). The completeness of the ONT assemblies also stabilized despite an increase in the ONT read depth, around ~92% and ~98% for Flye and Medaka assemblies.

Discussion

In this study, we have shown that near-complete genomes can be retrieved by multiplexing 96 bacterial isolates in the same long-read sequencing run. Despite an uneven distribution of ONT reads per barcode, we observed an average chromosomal contiguity of 0.89 which indicated that for most samples the chromosome was mostly represented by a single long contig. Furthermore, 92% of the plasmid sequences were circularized even for isolates with a low ONT read depth which makes this high-throughput multiplexing strategy an attractive choice for plasmid studies.

The availability of short-read sequencing data from the same bacterial isolates is still strongly desirable for three main reasons: (i) to perform an unbiased selection of isolates for long-read sequencing, (ii) to rely on the sequence accuracy achieved by short-read technologies (phred score > 30) and (iii) to include small plasmids underrepresented in the ONT library.

We proposed that given a short-read collection of isolates, the pangenome of these isolates can be computed and the presence/absence matrix of orthologous genes considered as the basis for the isolate selection. This collection should preferably include isolates with a low number of dead-ends (< 5) as this would ensure that the gene content can be confidently retrieved. Furthermore, a low number of dead-ends is crucial to obtain an optimal genome contiguity even if the obtained ONT coverage is low.

This selection ensures that complete genomes are generated from distinct clusters which are defined by the gene content of the isolates present in the collection. Furthermore, the generated complete genomes can be used to conduct reference-based approaches relying on the short-read data of the non-long read sequenced isolates belonging to a particular genomic cluster. A limitation of this approach is that the selected isolate may not possess other accessory genes present in clonally related isolates. This is particularly relevant for medium/small plasmids or phage elements consisting of only a few genes and thus having a small relative weight in the distance matrix used as a basis to assign the

clusters. Alternatively, choosing the isolates from the cluster with a highest distance to its centroid could select for isolates with a more distant gene content and potentially maximize the genome diversity. This issue would decrease if a higher number of centroids were selected since isolates with a more divergent gene content profile would be split into further sub-clusters during the k-means assignment.

The experimental setup used in this study (96 barcodes) and the uneven distribution of ONT reads per isolate makes it challenging to obtain full and high-quality genome sequences with only long-reads. Thus, the reported performance of Flye and Medaka is only informative considering the study constraints. We observed that an increase in the ONT read depth is critical to improve the accuracy of ONT-only assemblies, in particular for SNP calling. However, in the case of indels, the systematic and non-random ONT read errors reported in homopolymer sequences resulted in a high number of early terminated ORFs, even for the isolates with the highest ONT read depth (~ 150 ORFs). Given the uneven distribution of ONT reads observed in the multiplexing approach, the accuracy and completeness of ONT-only assemblies for the isolates with a low read depth can result in a high number of SNPs and indels in their complete genome sequences. This drawback still allows to identify the genomic context from a gene-of-interest (e.g AMR genes) but can limit studies based on SNP signatures such as outbreak investigations. Of note, the use of the newer Nanopore R10.3 chemistry with FLO-MIN111 flow cells would likely increase the base-pair accuracy of the ONT-only assemblies.

In the hybrid assemblies, the accuracy of the complete genomes is unaffected by the ONT read depth since, in general, the long-reads are only used as bridges to unequivocally connect short-read contigs. With fewer long-reads, we could still obtain a complete genome and its accuracy would be determined by the error read associated with the short-read technology. However, the accuracy of the hybrid assemblies can be affected by the quality of the initial short-read graph. If there is an elevated number of dead-ends in the short-read assembly graph, the sequence of ONT reads would be considered to complete the resulting genome and polishing that particular genomic region with Illumina reads would not be possible.

Recently, Dilthey et al. postulated a new method that allows skipping the multiplexing step by pooling together multiple non-barcoded samples in the same ONT flow cell [17]. This method relies on the availability of Illumina data and inter-sample genetic differences to *in silico* assign ONT reads to particular isolates. This strategy could reduce even further the sequencing costs per isolate and labour time associated with the molecular barcoding preparation. However, a current limitation of the tool is imposed by the high computational requirements required to perform the *in silico* assignment which can limit its applicability when a large number of samples (e.g. 96) are pooled in the same flow cell.

As previously reported [42], we also observed an underrepresentation of small plasmids in the ONT library which resulted in their absence in ONT-only assemblies. These plasmids can carry AMR or virulence genes and overlooking their presence in ONT-only assemblies when using ligation kits can affect subsequent analyses [42]. Small plasmids, however, are present in the initial short-read graph, usually as single circular contigs, and thus the absence of ONT reads covering these replicons do not affect the true representation of the genome. Ideally, the same genomic DNA would be used for both short- and long-read sequencing to avoid any potential bias and losing any plasmids due to the growth and extraction procedures used. In this study, a short-read data set previously created by Gladstone et al. [28] was used and a renewed growth and DNA extraction step was thereby needed for the ONT sequencing. This limitation could affect the stability of plasmids and may partly contribute to the underrepresentation of some extrachromosomal elements in the ONT library. However, due to the similarity of the growth conditions used, we expect the potential bias to be minimal in our study.

In conclusion, we have shown the potential of using the recently released ONT nanopore barcoding kit for 96 bacterial isolates to recover near-complete genomes in combination with prior short-read sequencing data. We propose a long-read isolate selection based on the gene content to ensure that the resulting complete genomes span the diversity present in the collection. Finally, the possibility of generating complete genomes on a high-throughput basis will likely continue to significantly advance the field of microbial genomics.

Data availability

ONT and Illumina sequencing data are available through the ENA Bioprojects PRJEB45354 and PRJEB32059 respectively. For each ONT barcode, individual ONT and Illumina accessions are indicated in Table S1. ONT reads and assemblies are available through the following permanent Figshare datasets: ONT reads -https://doi.org/10.6084/m9.figshare.14778333; Unicycler assemblies - https://doi.org/10.6084/m9.figshare.14705979; Flye assemblies - https://doi.org/10.6084/m9.figshare.14706015; Medaka polished assemblies - https://doi.org/10.6084/m9.figshare.14706108. The isolate selection pipeline is available at .https://gitlab.com/sirarredondo/long_read_selection . An Rmarkdown document with the code and files required to reproduce the results presented in this manuscript is available at https://gitlab.com/sirarredondo/highthroughput_strategy

Additional Files

Table S1. Summary of the ONT read statistics and ENA read accessions.

Table S2. Assembly statistics and mlplasmids prediction of the components present in the Unicycler assembly graph.

Table S3. Assembly statistics and mlplasmids prediction of the components present in the Flye assembly graph.

Abbreviations

AMR: Antimicrobial Resistance; INDEL: Insertions and deletions; IS: Insertion Sequence; ONT: Oxford Nanopore Technologies; ORF: Open-Reading-Frame; SNP: Single-Nucleotide Polymorphism; WGS: Whole-Genome Sequencing

Author's contributions

PJJ, ØS and JC designed and sought funding for the study. SA-A, AS and JC designed and implemented the isolate selection pipeline. AKP and FC performed the HMW DNA extractions. SA-A performed the computational analyses: read processing, assembly and genome statistics. RAG facilitated the short-read sequencing data and assemblies, and provided the popPUNK lineages. SA-A, AKP and JC wrote the first draft of the manuscript. All authors contributed and reviewed the manuscript.

Competing Interests

The authors declare that they have no competing interests.

Funding

This project has received funding from the European Union's Horizon 2020 research and innovation programme under the Marie Skłodowska-Curie (grant number 801133 to SA-A and AKP). This work was funded by the Trond Mohn Foundation (grant identifier TMS2019TMT04 to AKP, RAG, ØS, PJJ and JC). This work has been supported by the European Research Council (grant number 742158 to JC). It was also partially supported by the Joint Programming Initiative in Antimicrobial Resistance [JPIAMR Third call, STARCS, JPIAMR2016-AC16/00039]

Acknowledgments

We thank Lars Håland and Christopher Fenton at the Genomics Support Center Tromsø for generating, basecalling and demultiplexing the ONT sequencing reads.

References

1. Köser CU, Ellington MJ, Cartwright EJP, Gillespie SH, Brown NM, Farrington M, et al.. Routine use of microbial whole genome sequencing in diagnostic and public health microbiology. *PLoS Pathog*. 8:e10028242012;

2. Köser CU, Holden MTG, Ellington MJ, Cartwright EJP, Brown NM, Ogilvy-Stuart AL, et al.. Rapid whole-genome sequencing for investigation of a neonatal MRSA outbreak. *N Engl J Med*. 366:2267–752012;

3. Hendriksen RS, Bortolaia V, Tate H, Tyson GH, Aarestrup FM, McDermott PF. Using Genomics to Track Global Antimicrobial Resistance. *Front Public Health*. 7:2422019;

4. Schürch AC, van Schaik W. Challenges and opportunities for whole-genome sequencing-based surveillance of antibiotic resistance. *Annals of the New York Academy of Sciences*. 1388:108–202017;

5. Didelot X, Bowden R, Wilson DJ, Peto TEA, Crook DW. Transforming clinical microbiology with bacterial genome sequencing. *Nat Rev Genet*. 13:601–122012;

6. Robinson ER, Walker TM, Pallen MJ. Genomics and outbreak investigation: from sequence to consequence. *Genome Med*. 5:362013;

7. Arredondo-Alonso S, Willems RJ, van Schaik W, Schürch AC. On the (im)possibility of reconstructing plasmids from whole-genome short-read sequencing data. *Microbial Genomics*. Microbiology Society; 2017; doi: 10.1099/mgen.0.000128.

8. Orlek A, Stoesser N, Anjum MF, Doumith M, Ellington MJ, Peto T, et al.. Plasmid Classification in an Era of Whole-Genome Sequencing: Application in Studies of Antibiotic Resistance Epidemiology. *Front Microbiol*. Frontiers; 8:1822017;

9. Amarasinghe SL, Su S, Dong X, Zappia L, Ritchie ME, Gouil Q. Opportunities and challenges in long-read sequencing data analysis. *Genome Biol*. 21:302020;

10. Nanopore Sequencing Accuracy. https://nanoporetech.com/accuracy Accessed 2021 Apr 28.

11. Loman NJ, Quick J, Simpson JT. A complete bacterial genome assembled de novo using only nanopore sequencing data. *Nat Methods*. Nature Publishing Group; 12:733–52015;

12. Risse J, Thomson M, Patrick S, Blakely G, Koutsovoulos G, Blaxter M, et al.. A single chromosome assembly of Bacteroides fragilis strain BE1 from Illumina and MinION nanopore sequencing data. *Gigascience*. 4:602015;

13. Watson M, Warr A. Errors in long-read assemblies can critically affect protein prediction. Nat. Biotechnol. p. 124–6.

14. Medaka: Sequence correction provided by ONT Research. https://github.com/nanoporetech/medaka Accessed 2021 Sep 21.

15. De Maio N, Shaw LP, Hubbard A, George S, Sanderson ND, Swann J, et al.. Comparison of long-read sequencing technologies in the hybrid assembly of complex bacterial genomes. *Microb Genom*. 2019; doi: 10.1099/mgen.0.000294.

16. Wick RR, Judd LM, Gorrie CL, Holt KE. Completing bacterial genome assemblies with multiplex MinION sequencing. *Microbial Genomics*. Microbiology Society; 2017; doi: 10.1099/mgen.0.000132.

17. Dilthey AT, Meyer SA, Kaasch AJ. Ultraplexing: increasing the efficiency of long-read sequencing for hybrid assembly with k-mer-based multiplexing. *Genome Biol*. 21:682020;

18. Lipworth S, Pickford H, Sanderson N, Chau KK, Kavanagh J, Barker L, et al.. Optimized use of Oxford Nanopore flowcells for hybrid assemblies. *Microb Genom*. 2020; doi: 10.1099/mgen.0.000453.

19. Wick RR, Judd LM, Gorrie CL, Holt KE. Unicycler: Resolving bacterial genome assemblies from short and long sequencing reads. *PLoS Comput Biol*. Public Library of Science; 13:e10055952017;

20. Nguyen SH, Cao MD, Coin LJM. Real-time resolution of short-read assembly graph using ONT long reads. *PLoS Comput Biol*. 17:e10085862021;

21. Arredondo-Alonso S, Top J, McNally A, Puranen S, Pesonen M, Pensar J, et al.. Plasmids Shaped the Recent Emergence of the Major Nosocomial Pathogen *Enterococcus faecium*. *MBio*. 2020; doi: 10.1128/mBio.03284-19.

22. Pöntinen AK, Top J, Arredondo-Alonso S, Tonkin-Hill G, Freitas AR, Novais C, et al.. Apparent nosocomial adaptation of *Enterococcus faecalis* predates the modern hospital era. *Nat Commun*. 12:15232021;

23. Page AJ, Cummins CA, Hunt M, Wong VK, Reuter S, Holden MTG, et al.. Roary: rapid large-scale prokaryote pan genome analysis. *Bioinformatics*. 31:3691–32015;

24. Tonkin-Hill G, MacAlasdair N, Ruis C, Weimann A, Horesh G, Lees JA, et al.. Producing polished prokaryotic pangenomes with the Panaroo pipeline. *Genome Biol*. 21:1802020;

25. Maaten L van der, Hinton G. Visualizing Data using t-SNE. *J Mach Learn Res*. 9:2579–6052008;

26. Kanungo T, Mount DM, Netanyahu NS, Piatko CD, Silverman R, Wu AY. An efficient k-means clustering algorithm: analysis and implementation. *IEEE Trans Pattern Anal Mach Intell*. 24:881–922002;

27. Köster J, Rahmann S. Snakemake—a scalable bioinformatics workflow engine. *Bioinformatics*. Narnia; 28:2520–22012;

28. Gladstone RA, McNally A, Pöntinen AK, Tonkin-Hill G, Lees JA, Skytén K, et al.. Emergence and Dissemination of Antimicrobial Resistance in *Escherichia Coli* Causing Bloodstream Infections: A Nationwide Longitudinal Microbial Population Genomic Cohort Study in Norway between 2002-2017; 2:e331-e341;

29. Zerbino DR, Birney E. Velvet: algorithms for de novo short read assembly using de Bruijn graphs. *Genome Res*. 18:821–92008;

30. Boetzer M, Henkel CV, Jansen HJ, Butler D, Pirovano W. Scaffolding pre-assembled contigs using SSPACE. *Bioinformatics*. 27:578–92011;

31. Boetzer M, Pirovano W. Toward almost closed genomes with GapFiller. *Genome Biol*. 13:R562012;

32. Lees JA, Harris SR, Tonkin-Hill G, Gladstone RA, Lo SW, Weiser JN, et al.. Fast and flexible bacterial genomic epidemiology with PopPUNK. *Genome Res*. 29:304–162019;

33. Arredondo-Alonso S, Rogers MRC, Braat JC, Verschuuren TD, Top J, Corander J, et al.. mlplasmids: a user-friendly tool to predict plasmid- and chromosome-derived sequences for single species. *Microb Genom*. 2018; doi: 10.1099/mgen.0.000224.

34. Langmead B, Salzberg SL. Fast gapped-read alignment with Bowtie 2. *Nat Methods*. 9:357–92012;

35. Li H, Durbin R. Fast and accurate short read alignment with Burrows-Wheeler transform. *Bioinformatics*. 25:1754–602009;

36. Li H, Handsaker B, Wysoker A, Fennell T, Ruan J. 692 (2009). The Sequence Alignment/Map format and SAMtools. *Bioinformatics*. 25:2078–9.

37. Kolmogorov M, Yuan J, Lin Y, Pevzner PA. Assembly of long, error-prone reads using repeat graphs. *Nat Biotechnol*. 37:540–62019;

38. Gurevich A, Saveliev V, Vyahhi N, Tesler G. QUAST: Quality assessment tool for genome assemblies. *Bioinformatics*. 29:1072–52013;

39. Buchfink B, Xie C, Huson DH. Fast and sensitive protein alignment using DIAMOND. *Nat Methods*. 12:59–602015;

40. Altschul SF, Gish W, Miller W, Myers EW, Lipman DJ. Basic local alignment search tool. *J Mol Biol*. 215:403–101990;

41. Simão FA, Waterhouse RM, Ioannidis P, Kriventseva EV, Zdobnov EM. BUSCO: assessing genome assembly and annotation completeness with single-copy orthologs. *Bioinformatics*. 31:3210–22015;

42. Wick RR, Judd LM, Wyres KL, Holt KE. Recovery of small plasmid sequences via Oxford Nanopore sequencing. *Microb Genom*. 2021; doi: 10.1099/mgen.0.000631.

43. Million-Weaver S, Camps M. Mechanisms of plasmid segregation: have multicopy plasmids been overlooked? *Plasmid*. 75:27–362014;

Figure Legends

Figure 1. T-sne plot of the isolate selection, based on Jaccard distances computed from the presence/absence of genes defined by Panaroo in 3254 *E. coli* isolates. The five most predominant PopPUNK lineages are indicated with distinct colours, the rest of the lineages were merged into the category ‘Other’ (in yellow). To showcase the pipeline, we fixed the number of centroids to 96 which corresponded to the maximum number of isolates that can be multiplexed in the same MinION flow cell. The final coordinates of the centroids (n = 96) used in k-means are indicated as black diamond points. For each centroid (n = 96), the isolate closest to its respective centroid has been marked as ‘Selected’. In total, we indicate 96 *E. coli* isolates spanning the genomic diversity inherent in the collection that could be selected for further long-read sequencing.

Figure 2. Oxford Nanopore Technologies (ONT) statistics per barcode (n = 96). First panel: boxplot of the number of bases (Mbp) generated in the sequencing run. Second panel: boxplot of the N50

ONT read length (kbp). Third panel: boxplot of the phred score (log10 scale) associated with the ONT reads.

Figure 3. Unicycler statistics for the 96 isolates included in the ONT sequencing. Boxplots showing the component size (first panel) and contiguity values (second panel) together with an evaluation of the correlation between the number of ONT bases generated and the contiguity values achieved (third panel). A) Statistics for the chromosome component (largest component), the largest contig was confirmed as chromosome-derived with mlplasmids. B) Statistics for medium and large plasmid components (size > 10 kbp), the largest contig was confirmed as plasmid-derived with mlplasmids.

Figure 4. Illumina and ONT depth relative coverage of the plasmid components present in the Unicycler assemblies. The average depth of the plasmid replicons was normalized against the average depth of the chromosome to obtain a relative depth (log2 scale). Each plasmid is represented by two connected points depending on the sequencing technology.

Figure 5. Correlation between the number of ONT bases generated per isolate and genome accuracy statistics for Flye assemblies (green), Medaka-polished assemblies (orange) and Unicycler assemblies (purple). A) Number of SNPs per 100 kbp (log10 scale) computed considering as reference short-read contigs generated by an independent assembler. B) Number of indels per 100 kbp (log10 scale) computed considering as reference short-read contigs generated by an independent assembler. C) Number of early terminated ORFs based on the ideel test. D) Completeness of the assemblies as a percentage of complete single-copy conserved BUSCO genes (n = 440).

Figure S1. Histogram with the proportion of orthologous genes captured by the selected 96 *E. coli* isolates. In white, the total number of orthologous genes (n = 33508) present at the 3254 *E. coli* isolates were split into 100 bins based on their frequency. In green, the orthologous genes (n = 16308) present at the 96 selected isolates were split into the same 100 bins. For each white bin, we can observe the proportion of genes (in green) that would be recovered by sequencing the 96 *E. coli* isolates selected in Figure 1.

Figure S2. Genome accuracy comparison between SPAdes assemblies and Unicycler (hybrid) assemblies considering as reference short-read contigs assembled with Velvet. A) Number of SNP differences per 100 kbp observed in the SPAdes assemblies (black circles) and Unicycler assemblies (yellow circles). For each barcode, the difference in SNPs/100 kbp is represented by a line connecting the results of the two assemblies. B) Number of indel differences per 100 kbp observed in the SPAdes assemblies (black circles) and Unicycler assemblies (yellow circles). For each barcode, the difference in indels/100 kbp is represented by a line connecting the results of the two assemblies.

Figure S3. Overview of the hybrid assembly accuracy based on the quality of the initial SPAdes assembly graph in terms of dead-ends (x-axis). A) Number of SNP differences per 100 kbp in comparison to the number of dead-ends present in the SPAdes assembly graph. Each dot represents a barcode and its size and colour differs depending on the number of contigs (SR contigs) present at the SPAdes assembly graph. B) Number of indel differences per 100 kbp in comparison to the number of dead-ends present in the SPAdes assembly graph. Each dot represents a barcode and its size and colour differs depending on the number of contigs (SR contigs) present at the SPAdes assembly graph.

Figure1

Click here to access/download;Figure;Figure1.pdf

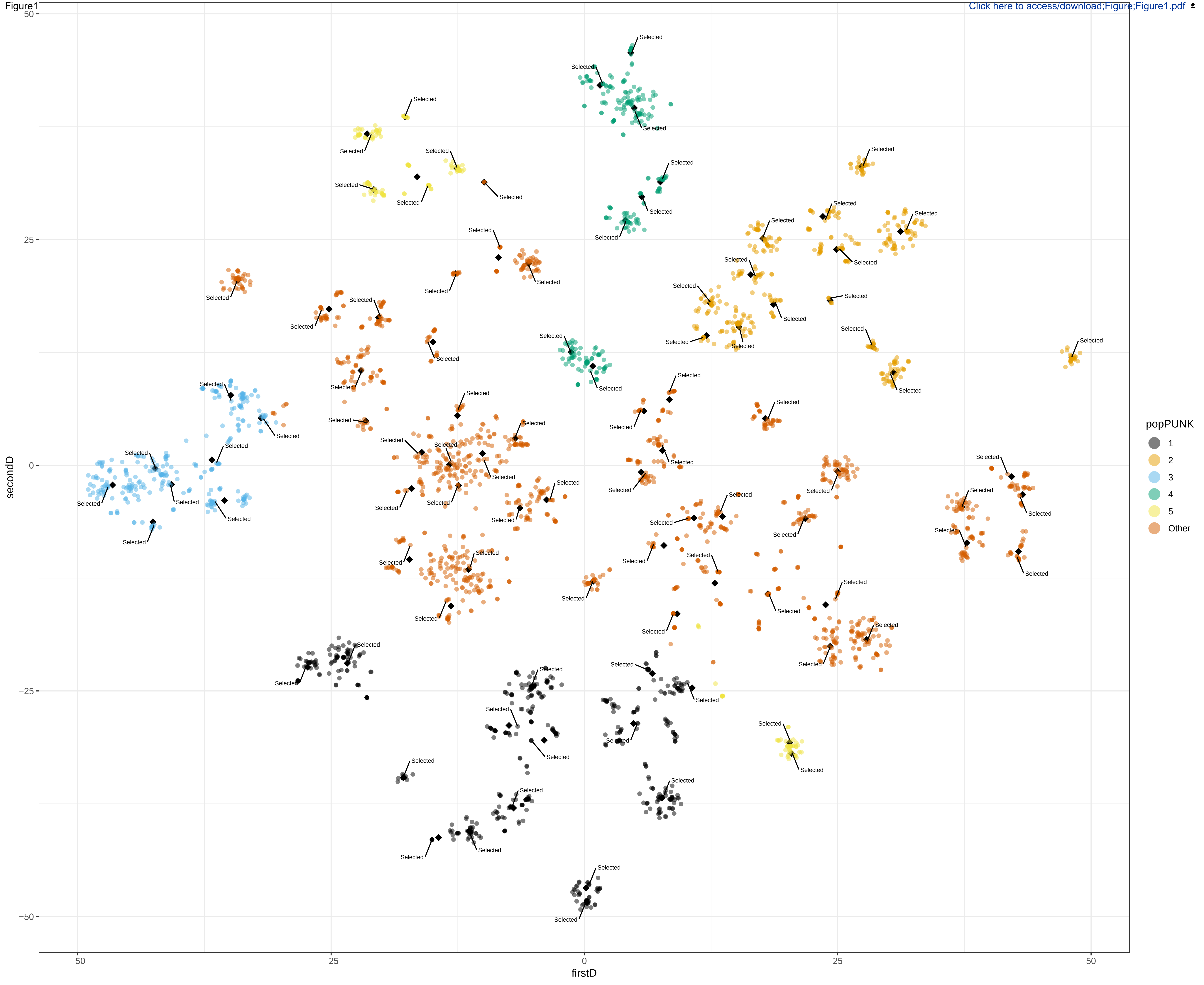

Figure2

Click here to access/download;Figure;Figure2.pdf

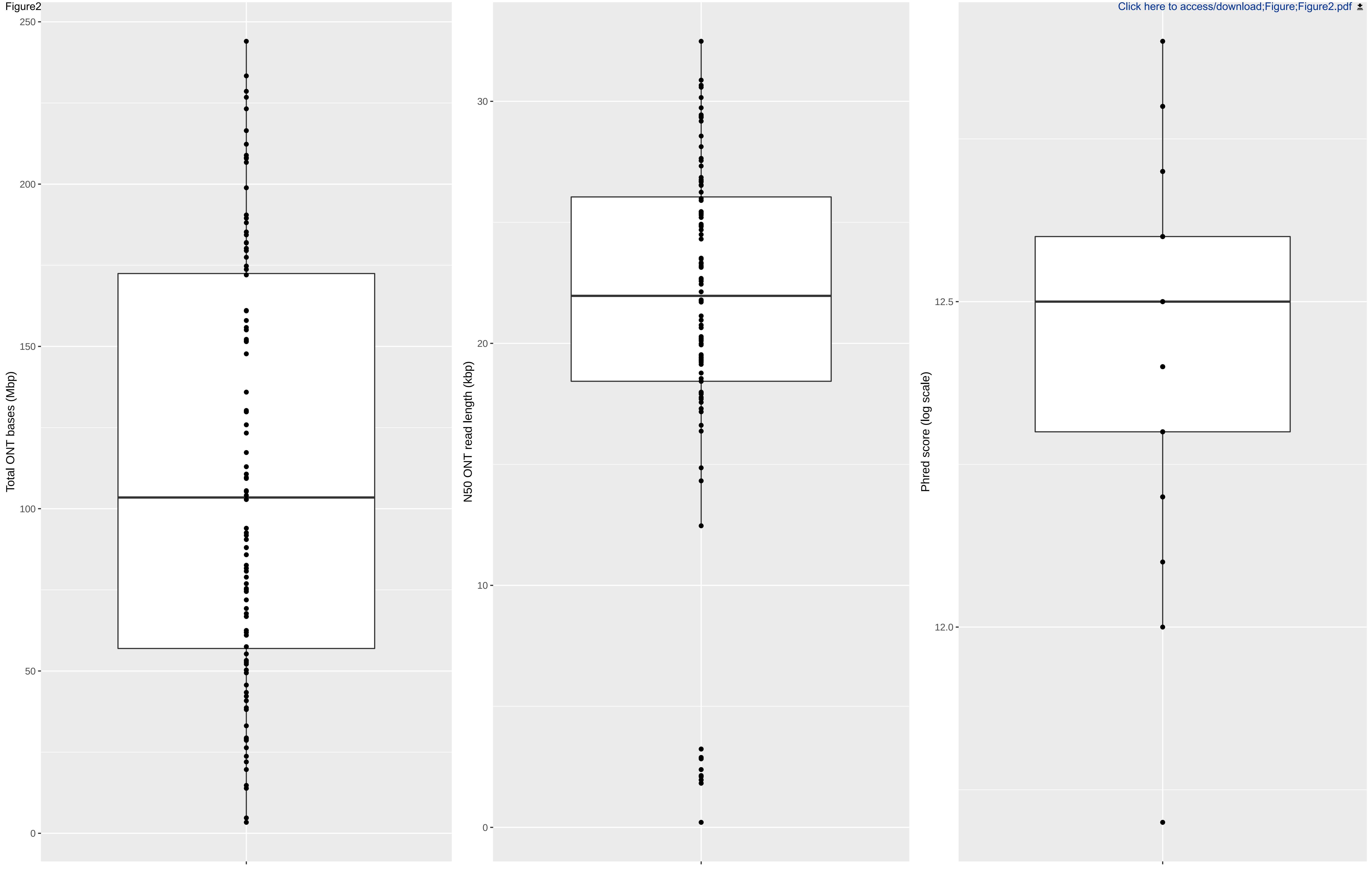

Figure3
Click here to access/download;Figure;Figure3.pdf

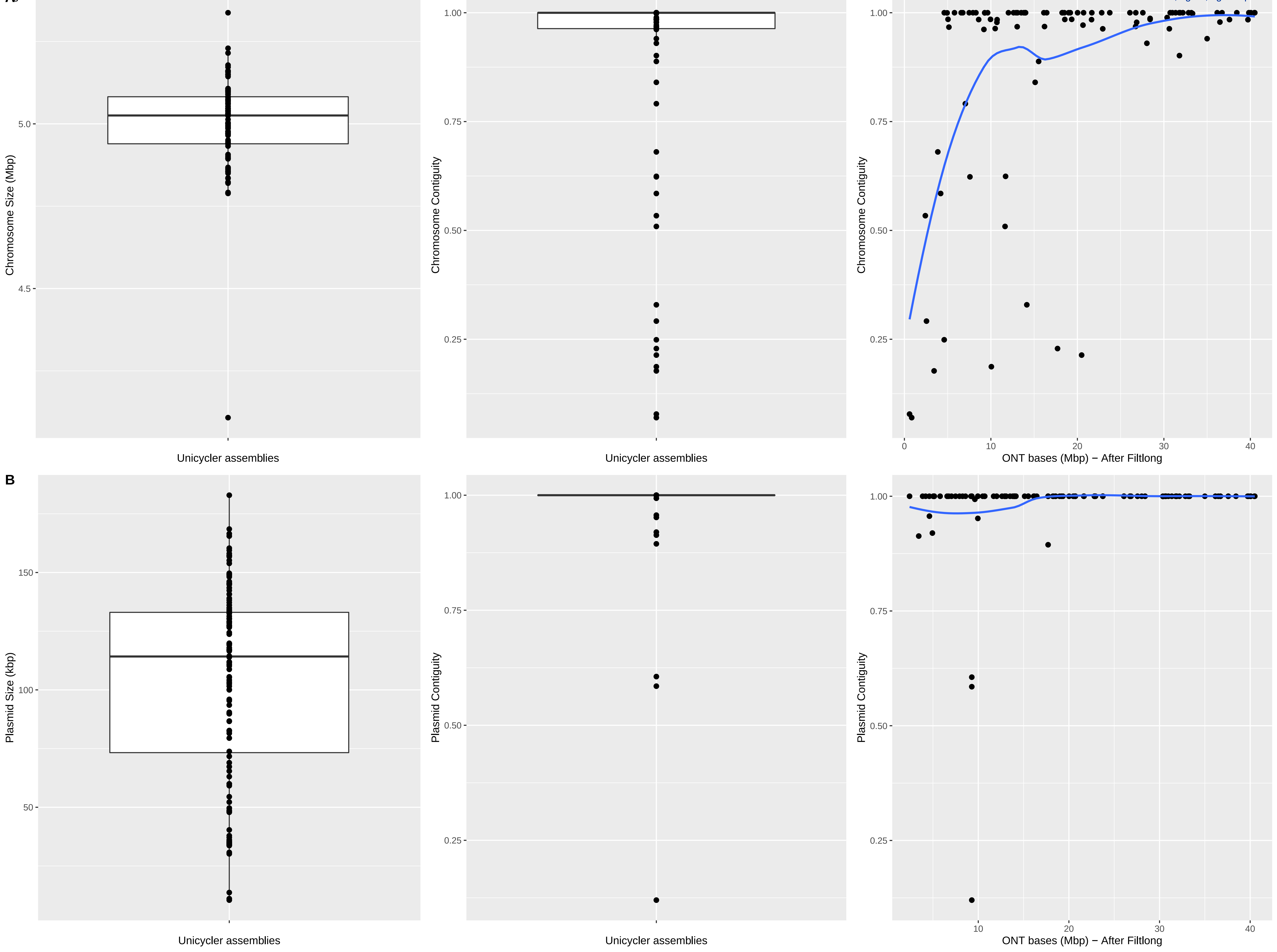

Figure4

Click here to access/download;Figure;Figure4.pdf

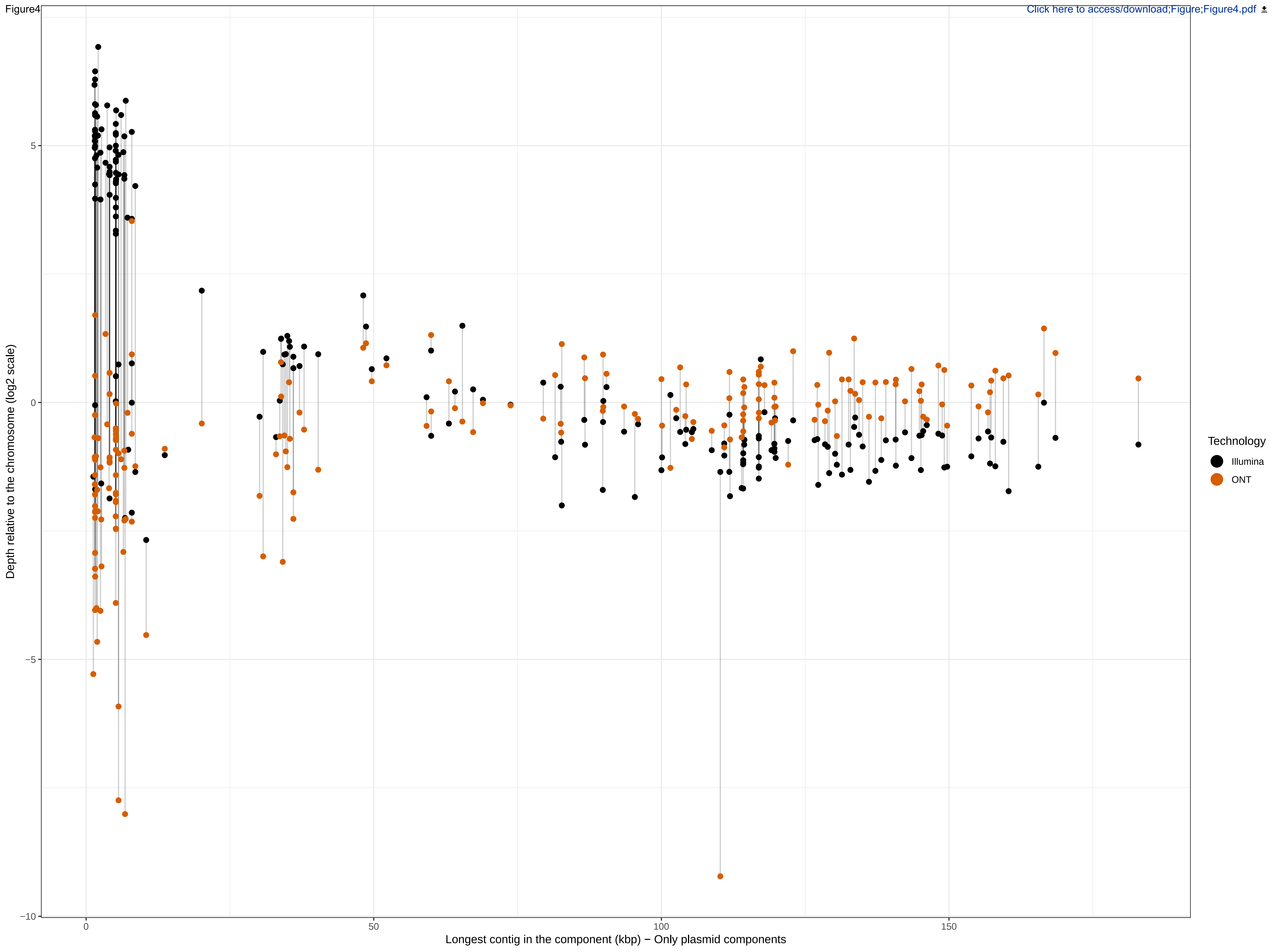

Figure5

Click here to access/download;Figure;Figure5.pdf

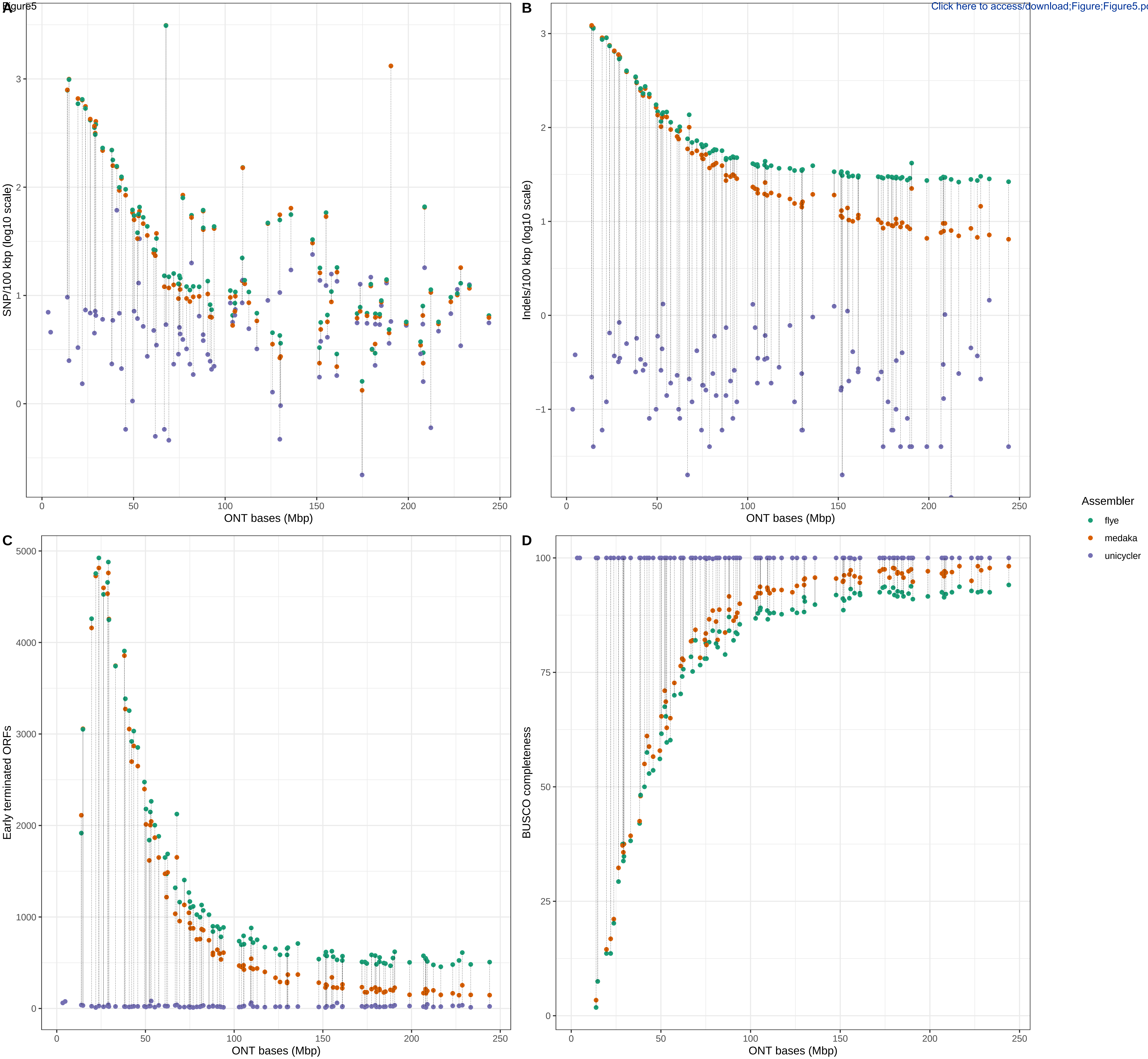

Additional FIle 1

Click here to access/download
Supplementary Material
Suppl_Table_S1.csv

Additional File 2

Click here to access/download
Supplementary Material
Suppl_Table_S2.csv

Additional File 3

Click here to access/download
Supplementary Material
Suppl_Table_S3.csv

FigureS1

Click here to access/download
Supplementary Material
FigureS1.pdf

FigureS2

Click here to access/download
Supplementary Material
FigureS2.pdf

FigureS3

Click here to access/download
Supplementary Material
FigureS3.pdf

Click here to access/download
Supplementary Material
MM_Revision_Track_Changes_Submission.docx
