# Supplementary figures and images for: A high-throughput multiplexing and selection strategy to complete bacterial genomes
Source: Gigascience. 2021 Dec 9;10(12):giab079. doi: 10.1093/gigascience/giab079 (PMC8673558; doi:10.1093/gigascience/giab079)

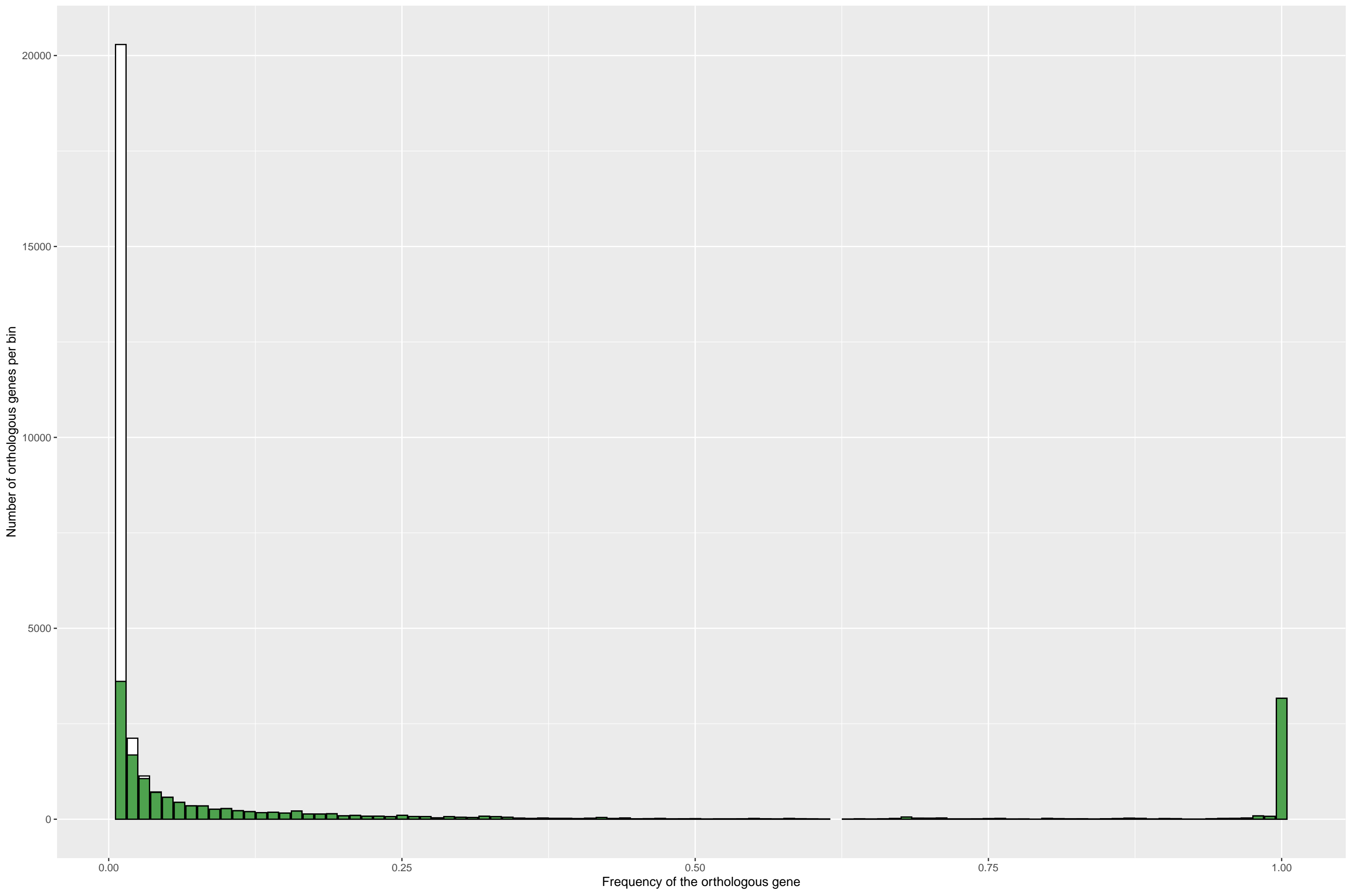

Supplement: giab079_Supplemental_Figures_and_Tables [file giab079_supplemental_figures_and_tables.zip › FigureS1.pdf]

A

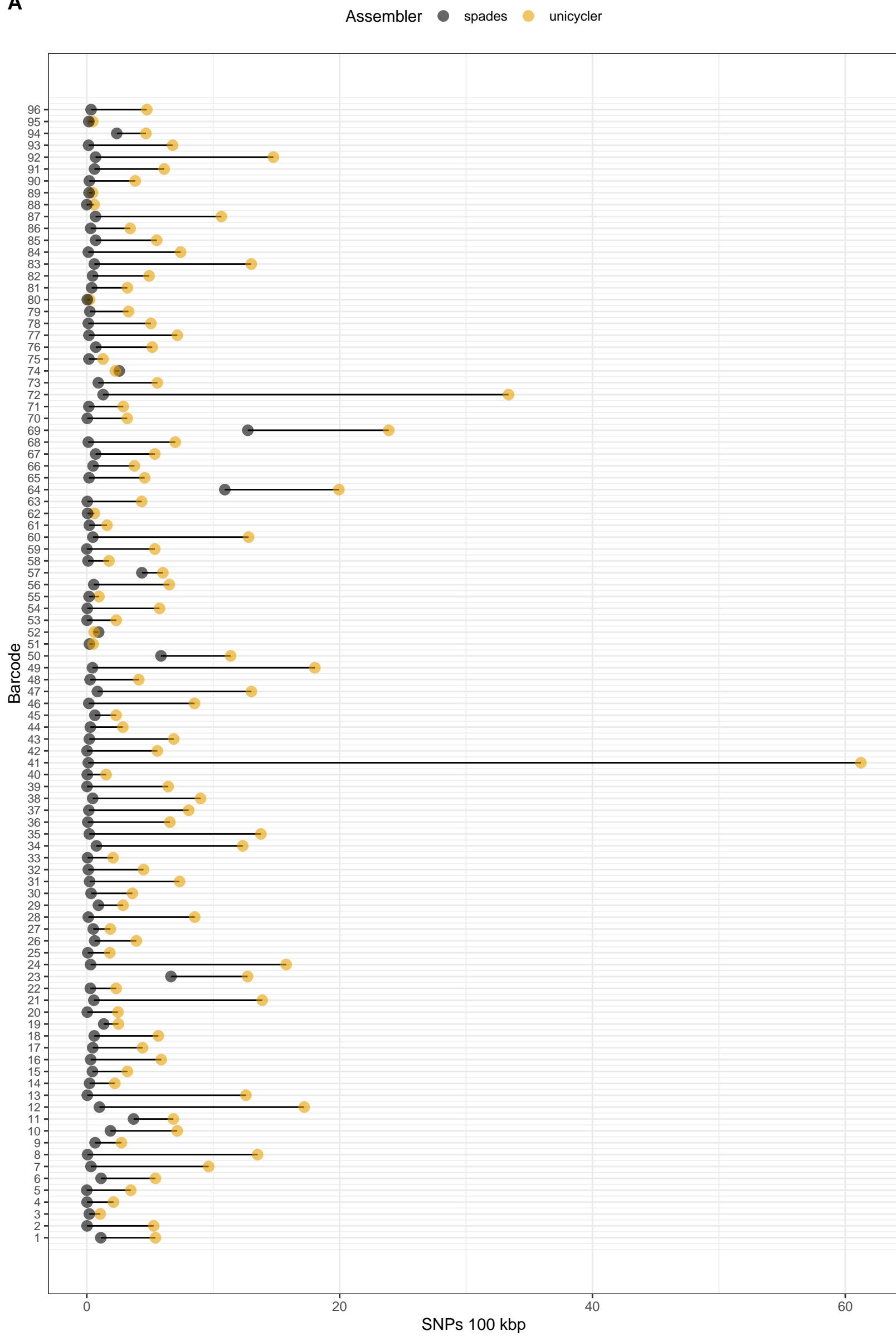

B

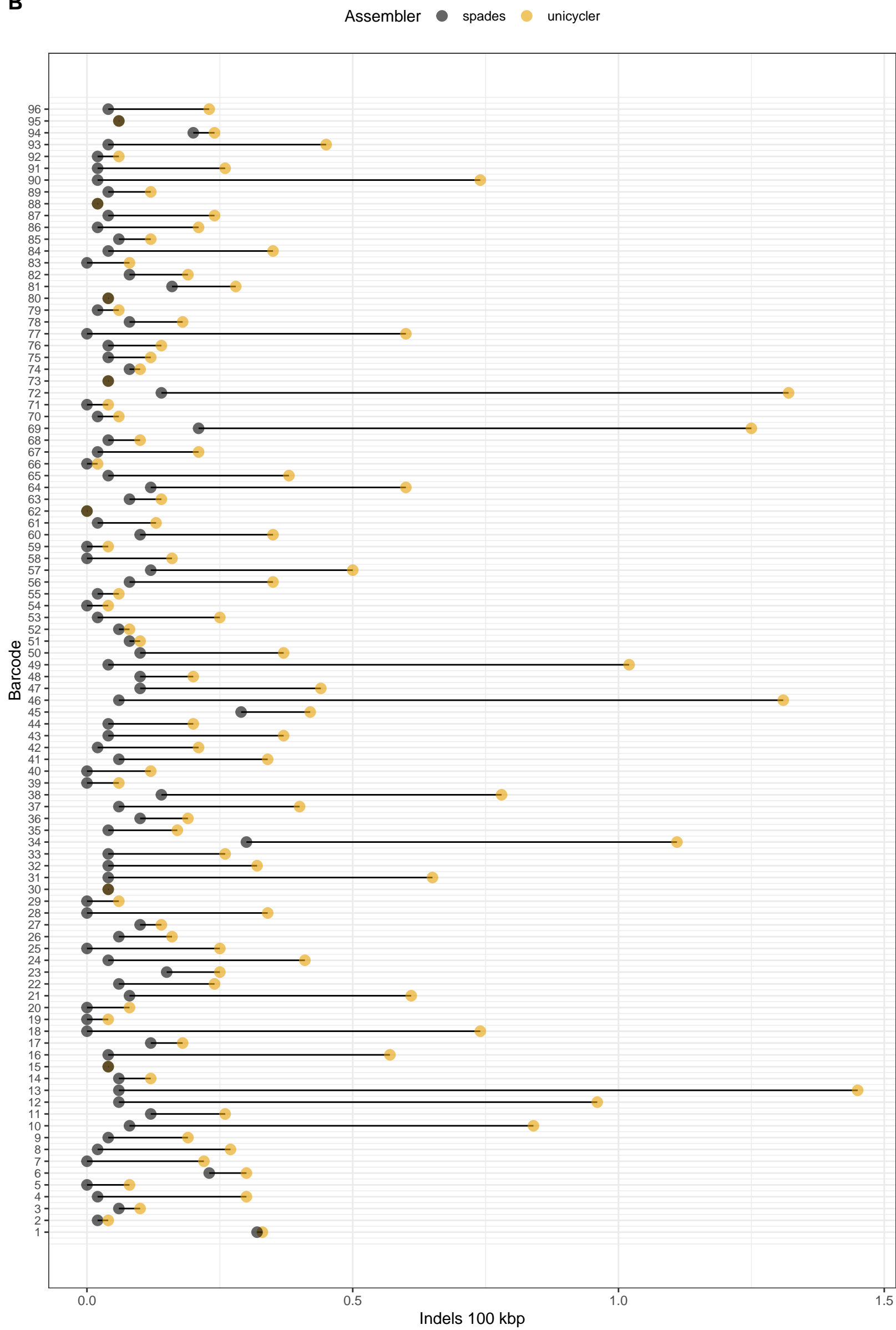

Supplement: giab079_Supplemental_Figures_and_Tables [file giab079_supplemental_figures_and_tables.zip › FigureS2.pdf]

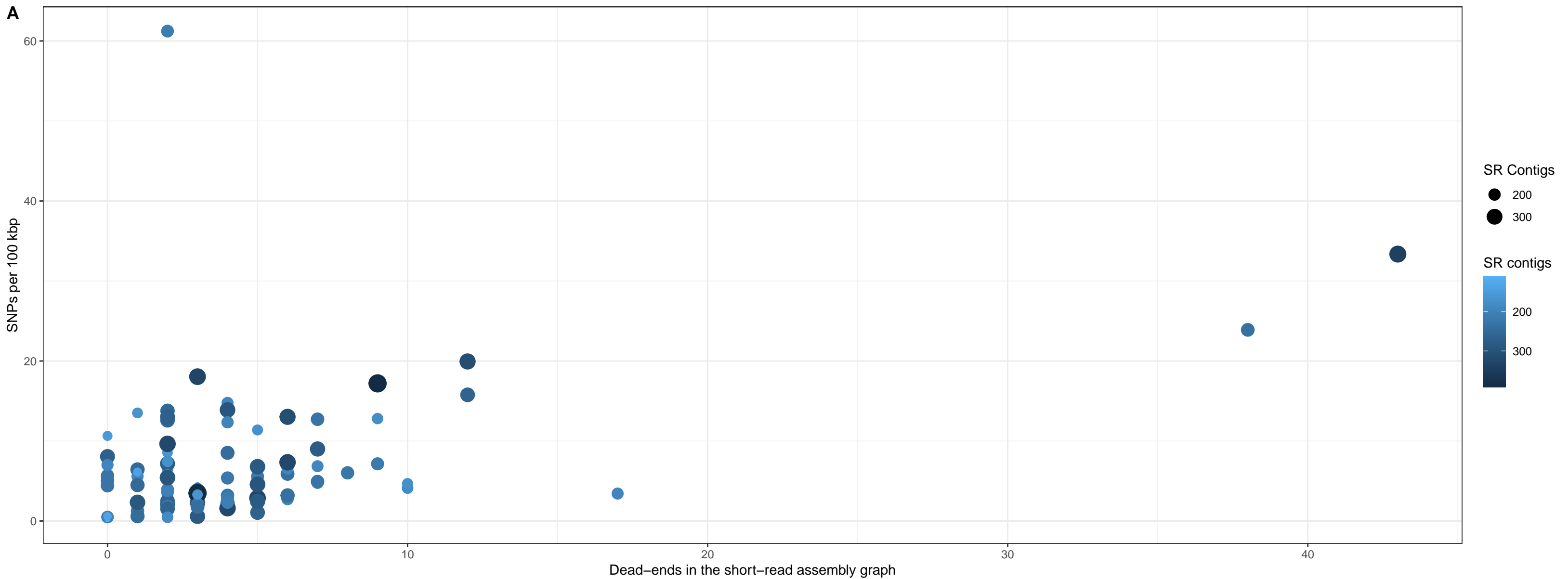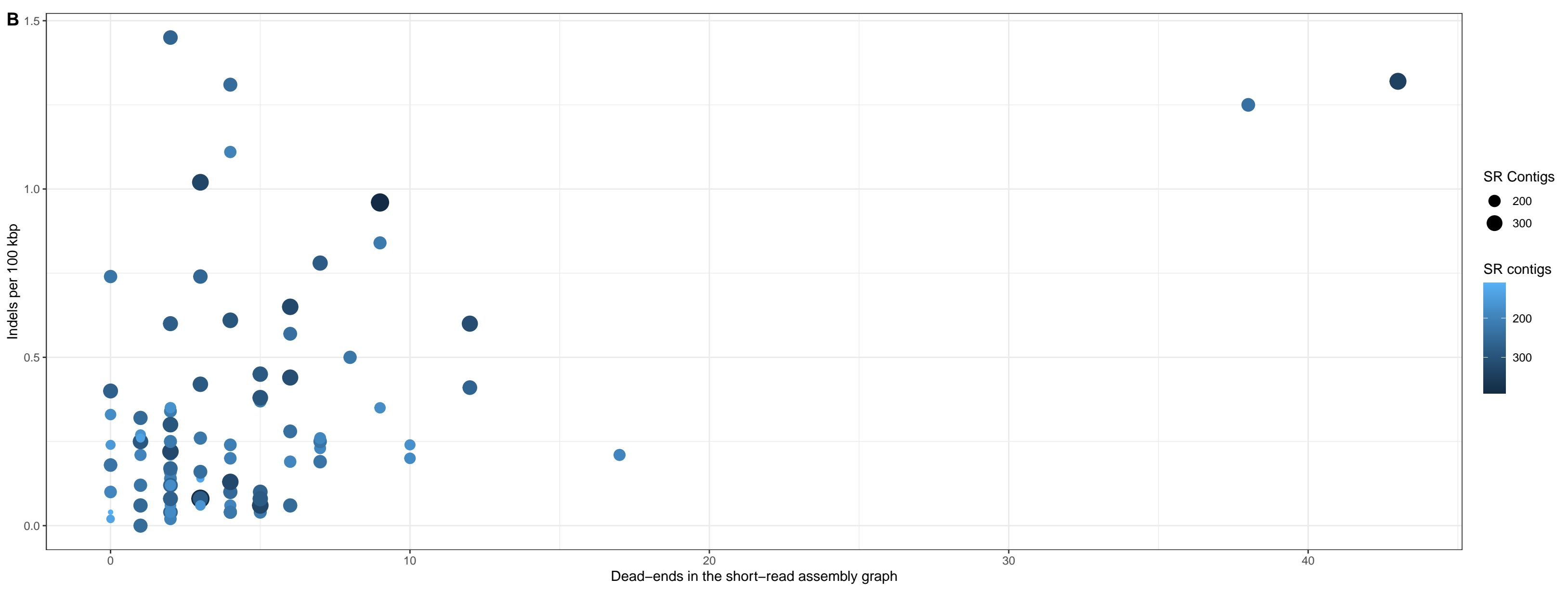

Supplement: giab079_Supplemental_Figures_and_Tables [file giab079_supplemental_figures_and_tables.zip › FigureS3.pdf]
