# Supplementary material for: A high-throughput multiplexing and selection strategy to complete bacterial genomes
Source: Gigascience. 2021 Dec 9;10(12):giab079. doi: 10.1093/gigascience/giab079 (PMC8673558; doi:10.1093/gigascience/giab079)
Supplement: giab079_Response_to_Reviewer_Comments_Original_Submission [file giab079_response_to_reviewer_comments_original_submission.pdf]

Author's Response To Reviewer Comments

Close

We thank the reviewers for their excellent comments and suggestions that have substantially increased the quality and clarity of the new revised version of the manuscript. In our response, we refer to the sections and page numbers from the track changes version of the manuscript.

Reviewer #1: In their manuscript, Arredondo-Alonso et al. provide an evaluation of the new 96 barcode kit from Oxford Nanopore, as well as some new methods to select a representative set of isolates for long-read sequencing based on preexisting short-read data. The paper is well-written and clear and the isolate selection method is interesting; the evaluation as well as the underlying dataset itself (which could be used for e.g. further methods development or benchmarking) will be of interest to the long-read sequencing community.

We have, however, a few remarks that we would recommend be addressed prior to the publication of the manuscript:

Major:

Q: If short-read data are already available, it is not clear that long-read barcoding is still necessary (as opposed to sequencing a barcode-less pool of high molecular weight DNA from the samples of interest, see https://genomebiology.biomedcentral.com/articles/10.1186/s13059-020-01974-9). This should at least be discussed - of course it would be interesting to see the performance of Ultraplexing on the generated dataset (simply ignoring the information present in the barcodes), but perhaps this is beyond the scope of this manuscript.

R: Thanks for pointing us to this excellent method to assign non-barcoded ONT sequences using preexisting short-read sequencing data. Unfortunately, we were unsuccessful in trying the tool on our set of 96 isolates due to the memory requirements of Ultraplexing (70 CPU hours and 175 GB of memory estimated for 48 samples). In our case, these requirements were even larger since we are dealing with 96 samples. We created a Singularity image to avoid installing a Perl dependency that could not be installed in our compiler version. However, despite trying Ultraplexer on two different HPC clusters, we could not allocate such a large amount of memory to the Singularity image that we created to run Ultraplexing.

Since comparing both methods was beyond the scope of the manuscript, which was mainly to benchmark the 96 barcoding kit from ONT, we decided only to discuss the potential pros/cons of using Ultraplexing in the Discussion of the manuscript (page 17).

We believe the method is fascinating and argue that this alternative can be attractive to reduce even more the ONT cost per isolate due to skipping the long-read barcoding step, especially if future releases of Ultraplexing permit to reduce the computational requirements.

Q: Evaluation of the long-read sample selection method. It is not entirely clear whether the method was applied to the collection collection of 3254 isolates or to a subset of 1085 isolates, and whether the number of centroids was set to 1085, or to 96 ("the number of centroids of the selection procedure were set to a large number of desired long-read isolates (n = 1085)"... but the number of centroids correspondes to the number of samples selected for long-read data generation, and actually sequenced were only 96 isolates?).

R: We thank the reviewers for pointing this out. We acknowledge this was not sufficiently clearly described in the main manuscript.

We applied the selection procedure twice on the collection of 3254 isolates:

1) In the Results section ‘An isolate selection spanning the genome diversity inherent in a short-read collection’, we show the potential of the selection approach by just selecting 96 isolates (96 centroids). This was an arbitrary number that we selected because it matched with the maximum number of isolates that can be simultaneously multiplexed in the same ONT flow cell. These 96 hypothetical selected isolates have never been long-read sequenced and thus are different from those presented in the later Results section.

2) As part of an ongoing project, we selected 1085 isolates from the collection of 3254 E. coli isolates using the selection approach described in the Results section ‘An isolate selection spanning the genome diversity inherent in a short-read collection’. From these 1085 selected isolates, we investigated different possibilities to complete all these genomes in a high-throughput manner. In the manuscript, we show the results of multiplexing 96 from these 1085 isolates in the same MinION flow cell. These 96 isolates are different from those hypothetically selected at the Results section ‘A long-read selection spanning the genome diversity inherent in a short-read collection’, since they were selected using a different number of centroids (96 vs 1085).

We have explicitly clarified this difference at the end of the Results section ‘An isolate selection spanning the genome diversity inherent in a short-read collection’ (pages 10, 11) and the Methods section ‘Collection of Illumina short-read assemblies’ (pages 6, 7).

Q: Furthermore, the authors report that their selection of isolates covers 99.9% of variation present in the tsne matrix - this is a relevant statistic, but it would be important to complement this with additional statistics on how well their selection captures variation in the underlying matrix of orthologous genes - e.g. it would be important to know how many (of the total considered set) orthologous genes are present in the selected set, potentially
broken down by frequency (e.g. showing a histogram over orthologous gene frequency on the X axis, and the proportion of genes in the corresponding bin represented in the selected set shown as an additional metric on the Y axis for each bin).

R: This is an outstanding comment. We have followed the suggestion of the reviewers of creating a histogram showing the frequency of the orthologous genes grouping the genes in bins (based on their relative frequency). For each bin (n = 100), we have coloured the proportion of genes of that bin that were selected in the 96 isolates. This histogram is available as the new Supplementary Figure S1. From this figure, it is clear that the 96 isolates cover all orthologous genes which are present at a frequency higher than 0.05 (from 4th to 100th bin) and only for those genes present at a frequency of 0.01 (first bin in the histogram) there is a significant proportion of genes not selected. The genes present at a frequency of 0.01 most likely correspond to either phage related genes or genes present in particular extrachromosomal elements which are not shared by all the isolates from a particular k-means cluster.

These new confirmatory results are now summarised in the Results section ‘An isolate selection spanning the genome diversity inherent in a short-read collection’ (page 10).

Q: Also, please provide explicit formulae for between_SS and tot_SS.

R: We have provided an explicit formula for between_SS and tot_SS in the Methods section ‘Isolate selection based on existing short-read assemblies’ (page 5).

Q: The sequencing here was carried out with FLO-MIN106 (R9.4) flow cells. The base pair accuracy of Nanopore-only assemblies based on data generated with FLO-MIN111 (R10) flow cells would likely have been higher. This should be mentioned e.g. in the Discussion.

R: Thanks for pointing this out. We have added this excellent remark in the Discussion (page 17).

Q: For the accuracy of the Nanopore-only assemblies: Empirically we have found that running multiple rounds of Medaka polishing can improve the quality of the assemblies. Can the authors check on a subset of samples that this is not the case for the assemblies reported on here, or, if it is the case, also evaluate the accuracy of these assemblies after two rounds of polishing?

R: Thanks for this excellent comment. Following the suggestion, we performed a second round of Medaka polishing on all the genomes, and evaluated their accuracy in terms of SNPs, indels, interrupted ORFs and recovered BUSCO genes. For all metrics, the results were highly similar to the genomes obtained after a single round of Medaka polishing. We have included these new results in the Results

section ‘Genome accuracy and completeness of hybrid and ONT-only assemblies’ (pages 14,15).

Minor:

Q: Figure 5: In the legend, A and B are switched (in the figure itself, panel A shows SNPs and panel B INDELs). The authors could consider showing SNPs and INDELs on the log scale, as the differences in achieved accuracy between Flye-only, Flye+Medaka and Unicycler are hard to discern for higher coverages. The authors could also consider combining the Flye-only, Flye+Medaka and Unicycler plots (perhaps connecting the dots corresponding the same isolate with lines and using color to indicate assembly method) -- this type of visualization may give an even better idea of the per-isolate differences in accuracy.

R: We thank the reviewers for spotting the error in the legend of Figure 5, we have now addressed this (page 22).

Following the suggestions of the reviewers, we have decided to redraw Figure 5 in which we now compare SNPs and indels on a log scale, connecting with a dashed line the observations from the same isolate and using colors to differentiate the assemblers. For the number of early interrupted ORFs and recovered BUSCO genes, we have plotted the observations in the same manner but conserving their natural scale. This new visualization is excellent to observe that the number of SNPs is similar between ONT-only and hybrid assemblies when there is enough ONT data. However, for indels, early interrupted ORFs and recovered BUSCO genes there is a substantial difference between ONT-only and hybrid assemblies that cannot be addressed by increasing the coverage. This new figure nicely reflects these observations, we thank again the reviewers for this suggestion.

Q: Typo: Page 8, line 21. „idoneal" maybe should be „ideal".

R: Thanks for spotting this error. We have now changed the word.

Q: Typo: Page 5, line 21f. „...is available at as a https://gitlab.com/sirarredondo/long_read_selection Snakemake pipeline" should be „ is available as a Snakemake pipeline at https://gitlab.com/sirarredondo/long_read_selection".

R: Thanks for spotting this typo that occurred while editing the final version of the manuscript. We have now changed the sentence.

Q: Wording: Page 1, Background. „Bacterial whole-genome sequencing based on short-read sequencing data ...". "Sequencing based on sequencing data" is phrased strangely. Maybe „Bacterial whole-genome sequencing based on short-read technologies..." or „Bacterial whole-genome assemblies based on short-read sequencing data...".

R: We appreciate the suggestion, indeed the repetition of the word ‘sequencing’ was not optimal. We have followed the edit suggested and consider ‘Bacterial whole-genome sequencing based on short-read technologies’

Sebastian Alexander Fuchs & Alexander Dilthey
Reviewer #2: GIGA-D-21-00176 "A high-throughput multiplexing and selection strategy to complete bacterial genomes"

The study by Arredondo-Alonso et al. presents a strategy to select candidate bacterial isolates to be further subjected to long read sequencing in order to provide a complementary sequencing in addition to the already performed, short-read based sequencing of the genomes of those isolates.

I found the study well-structured, and interesting to read. My main comments concern the following points:
Q: A) Conceptually, the authors start from a collection of well-sequenced isolates based on short-read sequencing (SRS). They analyse the genomic diversity in the collection, cluster the isolates based on their difference in orthologous gene content, and select the desired number of isolates to be long-read sequenced based on isolates that are the best representative of "clusters" of diversity:
A1) Page 4. The start of the analysis is the matrix of orthologous genes (pangenome). One may argue that the isolates have already been sufficiently sequenced to obtain reliable estimate of gene content.

Otherwise the approach does not work. In that case, the advantage of long reads would mostly be to bridge the contigs, but not to discover new genes, etc. In case the SRS data is not of enough quality, the initial comparison of gene presence-absence would not work anymore. Thus, the authors should comment on what is the threshold to consider good or conversely, not suitable, SRS data to be used in their hybrid approach.

R: We thank the reviewer for this excellent comment. Indeed, as mentioned later in their review, the quality of SRS data is crucial to obtain a confident estimate of the gene content. For this reason, we believe that the best metric to indicate the completeness of the genome is the number of dead-ends present in the SRS assembly graph. At the same time, this is influenced by the SRS coverage obtained, in most cases, a low SRS coverage can result in a high number of dead-ends which can be translated as parts of the genome not sequenced/represented in the SRS graph. The number of dead-ends per genome can be retrieved using popular tools such as Bandage. In addition, as mentioned later by the reviewer, these dead-ends need to be completed with ONT reads which can result in an elevated number of SNPs and indels for that particular genomic region.

Based on these observations and supported by Figure S3, we have recommended and discouraged the use of SRS genomes with more than 5 dead-ends per genome (Discussion, page 16) which would indicate that several genomic regions are not present in the SRS genome. On the contrary, if SRS genomes with a low number of dead-ends were included, a reliable estimation of the gene content can be obtained.

Q: Page 3. The authors write "During the hybrid assembly process, only a fraction of the total number of long-reads generated are required to bridge and span the initial short-read assembly graph and thus a low ONT coverage is sufficient to complete a genome." Can the authors also cite some references for this sentence?

R: We thank the reviewer for pointing this out. We have added the following references to support this sentence:
Wick RR, Judd LM, Gorrie CL, Holt KE (2017) Unicycler: Resolving bacterial genome assemblies from short and long sequencing reads. PLOS Computational Biology 13(6): e1005595. https://doi.org/10.1371/journal.pcbi.1005595
Nguyen SH, Cao MD, Coin LJM (2021) Real-time resolution of short-read assembly graph using ONT long reads. PLOS Computational Biology 17(1): e1008586. https://doi.org/10.1371/journal.pcbi.1008586

Q: A2) Pages 4-5. Essentially, the authors apply k-means clustering to the isolate coordinates obtained from the t-SNE mapping of the genomic differences. It is thus not surprising that the k-means algorithm performs well (clustering data originating from an Euclidean space would indeed recover most of the variance by k-means clustering, because it also works in that space). The question is whether the k-means clustering could/should not rather be applied to the original (Ds,s) matrix. This would partition the original variation from gene content among isolates into distinct clusters. Otherwise the variance that is described is the variance in t-SNE mapping coordinates.

R: We thank the reviewer for this excellent comment. The main reason why we chose to apply the k-means clustering on the t-SNE mapping coordinates is that the algorithm works best with spherical clusters while it struggles with clusters of distinct shapes which could be present in the original matrix. The t-SNE algorithm ensures that the obtained clusters are spherical and thus are optimal to be searched with the k-means algorithm. We have now clarified in the Methods section 'Long-read selection based on existing short-read assemblies' (page 5) and Results section 'An isolate long-read selection spanning the genome diversity inherent in a short-read collection' (page 10) that the variance described refers to the variance of the t-SNE mapping rather than the variance of the original matrix.

Q: A3) The authors justifiably want to maximize the amount of genomic diversity they can recover from their collection by sequencing. Yet, their approach selects the most common genomic profile among isolates from a given cluster, so they maximize the chance of finding the same genes as mostly found in other isolates from the same k-means cluster. If the strategy truly is to maximize the genomic diversity, would more extreme or divergent profiles in a cluster be more interesting to sequence? Also the isolates with unusual parameters (low coverage, different GC%, a lot of accessory genes, many plasmids…) be more logical candidates to be first sequenced to expand the list of known genes in the collection?

R: This is an outstanding comment. As argued by the reviewer, in our proposed approach we select the

‘average’ isolate in each k-means cluster by retrieving the isolate with the lowest Euclidean distance to its cluster centroid. Isolates belonging to a particular k-means cluster carrying non-core genes only shared by a minority of the isolates in the cluster will not be selected since they will show a higher Euclidean distance to their centroid compared to other isolates in the cluster. Now, we mention in the Discussion (page 16) the possibility suggested by the reviewer of selecting isolates more distant to its centroid compared to other samples from the cluster with the goal of selecting isolates with a more extreme genome profile.

We believe this is a very important point and we have extended the discussion on this topic (see Discussion, page 16) and argued the pros/cons of the proposed selection approach. The main reason for not selecting first isolates with unusual parameters is that for downstream analyses, we rely on the reference genomes obtained. Thus, the selection of the ‘average’ isolate in the cluster represents best the gene content for that particular k-means cluster. Following this, the complete genomes resulting from isolates with a low SRS coverage could contain a higher number of SNPs and indels since these absent genomic regions should be completed with ONT reads. However, this could be circumvented by the excellent suggestion of the reviewer of using a threshold to exclude isolates with low coverage.

Furthermore, if the number of centroids is sufficiently high those isolates with unusual parameters will tend to form individual k-means clusters and will be selected whereas if only a few isolates can be selected for ONT sequencing, we argue that choosing those that represent the average gene content may benefit downstream analyses.

However, we strongly agree with the reviewer that other selection approaches may be more suitable depending on the ultimate goal for which the complete genomes are required.

Q: B) The authors claim that the majority of the 96 isolates were fully recovered by their approach (Page 3), but this may largely be due to the sequencing data obtained via short read sequencing. Indeed, with 10.71 Gbp (page 10) obtained for the 96 isolates together, this represents about 25x coverage assuming 4.5 MB per E. coli genome (assuming homogeneous coverage for normalized libraries). With this low coverage, the ONT data alone could not be enough to produce complete and high-quality genome sequences. Similarly, if the SRS data was of poor quality (say 10x sequenced), then the ONT data at this obtained coverage would not help much recovering full and high-quality genome sequences. Similarly, the evaluation of the assembly quality based on ONT data alone (Flye) pages 12-13 may not be fair in that context, because of the low sequencing depth imposed by the experimental study design.

R: We thank the reviewer for emphasizing these important points The ONT output (10.71 Gbp) is indeed a problem to obtain high-quality genomes only considering the long-reads obtained. During the evaluation of ONT-only assemblies, we aimed to show that with the imposed experimental setup the accuracy of the genomes with a lower ONT read depth is problematic. Thus, we have now emphasized in the Discussion (page 16) that the Flye results presented are heavily affected by the low ONT data obtained for some of the isolates due to an unequal distribution of ONT reads per barcode in the flow cell and that the assembler performs much better as shown in Figure 5 at isolates with high ONT data. Thus, as we report now in the Discussion, the reported performance of Flye and Medaka is only informative when considering the constraints imposed by our experimental setup.

In the same manner, the evaluation of the SRS data was explored in Figures S2 and Figures S3 to remark the importance of considering high-quality SRS data to obtain a full and high-quality genome. We have now added in the Discussion the importance of including isolates with a low number of dead-ends and indicated as 5 the optimal threshold to consider isolates for the long-read selection.

Q: C) The authors should indicate what was their expected coverage per isolate (page 6). Multiplexing 96 genomes on one flow cell would necessarily provide low coverage for many cases. Also Page 6, the authors seem not to have normalized the concentration of each library prior to sequencing to allow for more homogeneous representation of each isolate in the library.

R: Thanks for remarking this. We have now indicated in the Results section ’Uneven distribution of ONT reads in the 96 multiplexing approach’ (page 11) the expected genome coverage considering a genome size of 5Mbp (chromosome + extrachromosomal elements).

We have now indicated in the Methods section ‘ONT library preparation’ (page 7) that prior to library

preparation, the samples were adjusted to 400 ng to normalize the concentration of the samples in the library.

Q: D) Page 7 the authors wrote "a quality phred score of 20 (--mean_q_weight 20), retaining 90% of the total number of ONT reads (--keep_percent 90) from a maximum 40x coverage (--target_bases)." I doubt that any ONT read is left with such a hight Phred score! Please revise if necessary.

R: We thank the reviewer for spotting this error. Indeed, this flag from Filtlong https://github.com/rrwick/Filtlong does not correspond to the Phred score but a weight given by Filtlong to obtain a mean quality score. We have now changed this in the Methods section 'Hybrid assemblies' (page 7) with the explanation of the flag given by Filtlong.

Q: E) Plasmid presence. The authors did not indicate whether the same gDNA was used for both SRS and ONT sequencing. This may be of importance to judge whether a plasmid is present or not given a specific sequencing technology and assembly approach are used. The isolate may have lost its plasmid(s) due to culturing conditions etc. so the comparison may be biased.

R: This is an excellent point. We used different gDNA for the SRS and ONT sequencing due to the fact that the short-read collection was sequenced in a previous study (Gladstone et al. 2021, https://doi.org/10.1016/S2666-5247(21)00031-8). For this reason, we had to renew the growth of isolates and perform another DNA extraction step. As mentioned by the reviewer, this can introduce some bias when comparing extrachromosomal elements between ONT and Illumina libraries. We have now explicitly stated this limitation in the Discussion (page 17).

Q: F) Page 15 (Discussion). The authors wrote "In the hybrid assemblies, the accuracy of the complete genomes is unaffected by the ONT read depth since, in general, the long-reads are only used as bridges to unequivocally connect short-read contigs". This all depends on how good the assemblies based on SRS data are. If many genomic regions are missing after SRS, the ONT data would be the only reads available to cover those missing regions, hence the error rate of ONT will apply to those regions.

R: Thanks for this excellent point. In the same paragraph, we mention that the assembly quality is dependent on the initial quality of the short-read graph. And also state, as mentioned by the reviewer, that the regions missing in the SRS (dead-ends) in the final sequence would rely on the ONT reads since there are no short-reads available.

Q: Spelling mistakes, suggested edits:
- Abstract. The sentence "we propose a long-read isolate selection strategy that optimizes a representative selection of isolates" is not clear. I don't recommend aggregating succinctly the terms in "long-read isolate selection strategy", as this leads to conceptual unclarity. The same is true at several other places in the manuscript, e.g. Page 3 "Long-read selection based on existing short-read assemblies", "From a large collection of short-read isolates". Page 5 "To showcase the proposed long-read selection"… Please check throughout the manuscript.

R: This is an excellent point. We have rephrased in the whole manuscript the combination of words 'long-read selection' for 'isolate selection' to improve the clarity of the text.

Q: Page 2: Add a reference to "These long-reads can typically span repeat elements in a bacterial genome producing a contiguous assembly consisting of single and circular contigs per replicon (chromosome and/or plasmids)."

R: Thanks for this remark. We have added the following references:

Loman, N., Quick, J. & Simpson, J. A complete bacterial genome assembled de novo using only nanopore sequencing data. Nat Methods 12, 733–735 (2015). https://doi.org/10.1038/nmeth.3444
Judith Risse, Marian Thomson, Sheila Patrick, Garry Blakely, Georgios Koutsovoulos, Mark Blaxter, Mick Watson, A single chromosome assembly of Bacteroides fragilis strain BE1 from Illumina and MinION nanopore sequencing data, GigaScience, Volume 4, Issue 1, December 2015, s13742–015–0101–6, https://doi.org/10.1186/s13742-015-0101-6

Q: Page 2: Last sentence. The authors do not mention existing software that address the basecalling issues in ONT data (e.g. nanopolish, medaka) and significantly enhance the quality of the consensus sequences, based on ONT data alone.

R: Excellent point. We have added a sentence in this paragraph to explain that an improved consensus sequence can be generated using these two popular tools (Nanopolish and Medaka).

Q: Page 7: Please rephrase "To map the Illumina reads against each of the nucleotides assembled by Unicycler".

R: Thanks for this remark. We have rephrased this sentence.

Q: Page 8: "idoneal"?

R: Thanks for spotting this error. We have now used the word 'ideal'.

Close
