# Supplementary material for: A high-throughput multiplexing and selection strategy to complete bacterial genomes
Source: Gigascience. 2021 Dec 9;10(12):giab079. doi: 10.1093/gigascience/giab079 (PMC8673558; doi:10.1093/gigascience/giab079)
Supplement: giab079_Response_to_Reviewer_Comments_Revision_1 [file giab079_response_to_reviewer_comments_revision_1.pdf]

Author's Response To Reviewer Comments

Close

We thank the reviewers for their positive feedback and for actively contributing to improving the quality of the manuscript. In this revision, we have addressed the remaining comments.

Q: Could you check mathematical notation on pages 4 and 5? E.g., under (4), c is defined as the number of centroids, but under (5), it seems to stand for the coordinates of an individual centroid. Also, is it possible that norm signs are missing sometimes, e.g. in the definition of V(C_k)? t_i seems to be a two-dimensional variable (position within the t-sne projection?), so it is not exactly clear how to interpret the difference within the squared term.

A: We thank the reviewers for spotting and checking the mathematical notation. We have reformulated some of the formulas, rephrased some of the text and defined L as the number of centroids to avoid using the same notation for the individual position of a centroid in the t-sne coordinates. The changes are included on pages 4 and 5 of the manuscript (Methods section 'Isolate selection based on existing short-read assemblies').

Q: We have noticed a few typos: "onto flow cells.*.*" (page 7); "genomeassembled" (page 8); „and indicating the read*y* type" (page 8).

A: We thank the reviewers for spotting this. We have gone through the main manuscript and corrected all the remaining typos.

Close
