# Supplementary material for: A high-throughput multiplexing and selection strategy to complete bacterial genomes
Source: Gigascience. 2021 Dec 9;10(12):giab079. doi: 10.1093/gigascience/giab079 (PMC8673558; doi:10.1093/gigascience/giab079)
Supplement: giab079_Reviewer_1_Report_Original_Submission — Alexander Dilthey -- 8/4/2021 Reviewed [file giab079_reviewer_1_report_original_submission.pdf]

Reviewer Report

Title: A high-throughput multiplexing and selection strategy to complete bacterial genomes

Version: Original Submission **Date:** 8/4/2021

Reviewer name: Alexander Dilthey

Reviewer Comments to Author:

In their manuscript, Arredondo-Alonso et al. provide an evaluation of the new 96 barcode kit from Oxford Nanopore, as well as some new methods to select a representative set of isolates for long-read sequencing based on preexisting short-read data. The paper is well-written and clear and the isolate selection method is interesting; the evaluation as well as the underlying dataset itself (which could be used for e.g. further methods development or benchmarking) will be of interest to the long-read sequencing community.

We have, however, a few remarks that we would recommend be addressed prior to the publication of the manuscript:

Major:

- If short-read data are already available, it is not clear that long-read barcoding is still necessary (as opposed to sequencing a barcode-less pool of high molecular weight DNA from the samples of interest, see https://genomebiology.biomedcentral.com/articles/10.1186/s13059-020-01974-9). This should at least be discussed - of course it would be interesting to see the performance of Ultraplexing on the generated dataset (simply ignoring the information present in the barcodes), but perhaps this is beyond the scope of this manuscript.
- Evaluation of the long-read sample selection method. It is not entirely clear whether the method was applied to the collection collection of 3254 isolates or to a subset of 1085 isolates, and whether the number of centroids was set to 1085, or to 96 ("the number of centroids of the selection procedure were set to a large number of desired long-read isolates (n = 1085)"... but the number of centroids correspondes to the number of samples selected for long-read data generation, and actually sequenced were only 96 isolates?). Furthermore, the authors report that their selection of isolates covers 99.9% of variation present in the tsne matrix - this is a relevant statistic, but it would be important to complement this with additional statistics on how well their selection captures variation in the underlying matrix of orthologous genes - e.g. it would be important to know how many (of the total considered set) orthologous genes are present in the selected set, potentially broken down by frequency (e.g. showing a histogram over orthologous gene frequency on the X axis, and the proportion of genes in the corresponding bin represented in the selected set shown as an additional metric on the Y axis for each bin). Also, please provide explicit formulae for between_SS and tot_SS.
- The sequencing here was carried out with FLO-MIN106 (R9.4) flow cells. The base pair accuracy of Nanopore-only assemblies based on data generated with FLO-MIN111 (R10) flow cells would likely have been higher. This should be mentioned e.g. in the Discussion.
- For the accuracy of the Nanopore-only assemblies: Empirically we have found that running multiple rounds of Medaka polishing can improve the quality of the assemblies. Can the authors check on a

subset of samples that this is not the case for the assemblies reported on here, or, if it is the case, also evaluate the accuracy of these assemblies after two rounds of polishing?
Minor:
- Figure 5: In the legend, A and B are switched (in the figure itself, panel A shows SNPs and panel B INDELs). The authors could consider showing SNPs and INDELs on the log scale, as the differences in achieved accuracy between Flye-only, Flye+Medaka and Unicycler are hard to discern for higher coverages. The authors could also consider combining the Flye-only, Flye+Medaka and Unicycler plots (perhaps connecting the dots corresponding the same isolate with lines and using color to indicate assembly method) -- this type of visualization may give an even better idea of the per-isolate differences in accuracy.
- Typo: Page 8, line 21. â€židoneal" maybe should be â€žideal".
- Typo: Page 5, line 21f. â€ž...is available at as a https://gitlab.com/sirarredondo/long_read_selection Snakemake pipeline" should be â€ž is available as a Snakemake pipeline at https://gitlab.com/sirarredondo/long_read_selection".
- Wording: Page 1, Background. â€žBacterial whole-genome sequencing based on short-read sequencing data ...". "Sequencing based on sequencing data" is phrased strangely. Maybe â€žBacterial whole-genome sequencing based on short-read technologies..." or â€žBacterial whole-genome assemblies based on short-read sequencing data...".
Sebastian Alexander Fuchs & Alexander Dilthey

Methods

Are the methods appropriate to the aims of the study, are they well described, and are necessary controls included? Choose an item.

Conclusions

Are the conclusions adequately supported by the data shown? Choose an item.

Reporting Standards

Does the manuscript adhere to the journal's guidelines on minimum standards of reporting? Choose an item.

Choose an item.

Statistics

Are you able to assess all statistics in the manuscript, including the appropriateness of statistical tests used? Choose an item.

Quality of Written English

Please indicate the quality of language in the manuscript: Choose an item.

Declaration of Competing Interests

Please complete a declaration of competing interests, considering the following questions:

- Have you in the past five years received reimbursements, fees, funding, or salary from an organisation that may in any way gain or lose financially from the publication of this manuscript, either now or in the future?
- Do you hold any stocks or shares in an organisation that may in any way gain or lose financially from the publication of this manuscript, either now or in the future?
- Do you hold or are you currently applying for any patents relating to the content of the manuscript?
- Have you received reimbursements, fees, funding, or salary from an organization that holds or has applied for patents relating to the content of the manuscript?
- Do you have any other financial competing interests?
- Do you have any non-financial competing interests in relation to this paper?

If you can answer no to all of the above, write 'I declare that I have no competing interests' below. If your reply is yes to any, please give details below.

We declare we have no competing interests.

I agree to the open peer review policy of the journal. I understand that my name will be included on my report to the authors and, if the manuscript is accepted for publication, my named report including any attachments I upload will be posted on the website along with the authors' responses. I agree for my report to be made available under an Open Access Creative Commons CC-BY license (http://creativecommons.org/licenses/by/4.0/). I understand that any comments which I do not wish to be included in my named report can be included as confidential comments to the editors, which will not be published.

Choose an item.

To further support our reviewers, we have joined with Publons, where you can gain additional credit to further highlight your hard work (see: https://publons.com/journal/530/gigascience). On publication of this paper, your review will be automatically added to Publons, you can then choose whether or not to claim your Publons credit. I understand this statement.

Yes Choose an item.
