# Supplementary material for: A high-throughput multiplexing and selection strategy to complete bacterial genomes
Source: Gigascience. 2021 Dec 9;10(12):giab079. doi: 10.1093/gigascience/giab079 (PMC8673558; doi:10.1093/gigascience/giab079)
Supplement: giab079_Reviewer_2_Report_Original_Submission — Alban Ramette -- 8/10/2021 Reviewed [file giab079_reviewer_2_report_original_submission.pdf]

Reviewer Report

Title: A high-throughput multiplexing and selection strategy to complete bacterial genomes

Version: Original Submission **Date:** 8/10/2021

Reviewer name: Alban Ramette

Reviewer Comments to Author:

GIGA-D-21-00176 "A high-throughput multiplexing and selection strategy to complete bacterial genomes"
The study by Arredondo-Alonso et al. presents a strategy to select candidate bacterial isolates to be further subjected to long read sequencing in order to provide a complementary sequencing in addition to the already performed, short-read based sequencing of the genomes of those isolates.
I found the study well-structured, and interesting to read. My main comments concern the following points:
A) Conceptually, the authors start from a collection of well-sequenced isolates based on short-read sequencing (SRS). They analyse the genomic diversity in the collection, cluster the isolates based on their difference in orthologous gene content, and select the desired number of isolates to be long-read sequenced based on isolates that are the best representative of "clusters" of diversity:
A1) Page 4. The start of the analysis is the matrix of orthologous genes (pangenome). One may argue that the isolates have already been sufficiently sequenced to obtain reliable estimate of gene content. Otherwise the approach does not work. In that case, the advantage of long reads would mostly be to bridge the contigs, but not to discover new genes, etc. In case the SRS data is not of enough quality, the initial comparison of gene presence-absence would not work anymore. Thus, the authors should comment on what is the threshold to consider good or conversely, not suitable, SRS data to be used in their hybrid approach.
Page 3. The authors write "During the hybrid assembly process, only a fraction of the total number of long-reads generated are required to bridge and span the initial short-read assembly graph and thus a low ONT coverage is sufficient to complete a genome." Can the authors also cite some references for this sentence?
A2) Pages 4-5. Essentially, the authors apply k-means clustering to the isolate coordinates obtained from the t-SNE mapping of the genomic differences. It is thus not surprising that the k-means algorithm performs well (clustering data originating from an Euclidean space would indeed recover most of the variance by k-means clustering, because it also works in that space). The question is whether the k-means clustering could/should not rather be applied to the original (Ds,s) matrix. This would partition the original variation from gene content among isolates into distinct clusters. Otherwise the variance that is described is the variance in t-SNE mapping coordinates.
A3) The authors justifiably want to maximize the amount of genomic diversity they can recover from their collection by sequencing. Yet, their approach selects the most common genomic profile among isolates from a given cluster, so they maximize the chance of finding the same genes as mostly found in other isolates from the same k-means cluster. If the strategy truly is to maximize the genomic diversity,

would more extreme or divergent profiles in a cluster be more interesting to sequence? Also the isolates with unusual parameters (low coverage, different GC%, a lot of accessory genes, many plasmids…) be more logical candidates to be first sequenced to expand the list of known genes in the collection?

B) The authors claim that the majority of the 96 isolates were fully recovered by their approach (Page 3), but this may largely be due to the sequencing data obtained via short read sequencing. Indeed, with 10.71 Gbp (page 10) obtained for the 96 isolates together, this represents about 25x coverage assuming 4.5 MB per E. coli genome (assuming homogeneous coverage for normalized libraries). With this low coverage, the ONT data alone could not be enough to produce complete and high-quality genome sequences. Similarly, if the SRS data was of poor quality (say 10x sequenced), then the ONT data at this obtained coverage would not help much recovering full and high-quality genome sequences. Similarly, the evaluation of the assembly quality based on ONT data alone (Flye) pages 12-13 may not be fair in that context, because of the low sequencing depth imposed by the experimental study design.

C) The authors should indicate what was their expected coverage per isolate (page 6). Multiplexing 96 genomes on one flow cell would necessarily provide low coverage for many cases. Also Page 6, the authors seem not to have normalized the concentration of each library prior to sequencing to allow for more homogeneous representation of each isolate in the library.

D) Page 7 the authors wrote "a quality phred score of 20 (--mean_q_weight 20), retaining 90% of the total number of ONT reads (--keep_percent 90) from a maximum 40x coverage (--target_bases)." I doubt that any ONT read is left with such a hight Phred score! Please revise if necessary.

E) Plasmid presence. The authors did not indicate whether the same gDNA was used for both SRS and ONT sequencing. This may be of importance to judge whether a plasmid is present or not given a specific sequencing technology and assembly approach are used. The isolate may have lost its plasmid(s) due to culturing conditions etc. so the comparison may be biased.

F) Page 15 (Discussion). The authors wrote "In the hybrid assemblies, the accuracy of the complete genomes is unaffected by the ONT read depth since, in general, the long-reads are only used as bridges to unequivocally connect short-read contigs". This all depends on how good the assemblies based on SRS data are. If many genomic regions are missing after SRS, the ONT data would be the only reads available to cover those missing regions, hence the error rate of ONT will apply to those regions.

Spelling mistakes, suggested edits:

- Abstract. The sentence "we propose a long-read isolate selection strategy that optimizes a representative selection of isolates" is not clear. I don't recommend aggregating succinctly the terms in "long-read isolate selection strategy", as this leads to conceptual unclarity. The same is true at several other places in the manuscript, e.g. Page 3 "Long-read selection based on existing short-read assemblies", "From a large collection of short-read isolates". Page 5 "To showcase the proposed long-read selection"… Please check throughout the manuscript.
-Page 2: Add a reference to "These long-reads can typically span repeat elements in a bacterial genome producing a contiguous assembly consisting of single and circular contigs per replicon (chromosome and/or plasmids)."
-Page 2: Last sentence. The authors do not mention existing software that address the basecalling issues in ONT data (e.g. nanopolish, medaka) and significantly enhance the quality of the consensus sequences, based on ONT data alone.

-Page 7: Please rephrase "To map the Illumina reads against each of the nucleotides assembled by Unicycler".
-Page 8: "idoneal"?

Methods

Are the methods appropriate to the aims of the study, are they well described, and are necessary controls included? Choose an item.

Conclusions

Are the conclusions adequately supported by the data shown? Choose an item.

Reporting Standards

Does the manuscript adhere to the journal's guidelines on minimum standards of reporting? Choose an item.

Choose an item.

Statistics

Are you able to assess all statistics in the manuscript, including the appropriateness of statistical tests used? Choose an item.

Quality of Written English

Please indicate the quality of language in the manuscript: Choose an item.

Declaration of Competing Interests

Please complete a declaration of competing interests, considering the following questions:

- Have you in the past five years received reimbursements, fees, funding, or salary from an organisation that may in any way gain or lose financially from the publication of this manuscript, either now or in the future?
- Do you hold any stocks or shares in an organisation that may in any way gain or lose financially from the publication of this manuscript, either now or in the future?
- Do you hold or are you currently applying for any patents relating to the content of the manuscript?
- Have you received reimbursements, fees, funding, or salary from an organization that holds or has applied for patents relating to the content of the manuscript?
- Do you have any other financial competing interests?
- Do you have any non-financial competing interests in relation to this paper?

If you can answer no to all of the above, write 'I declare that I have no competing interests' below. If your reply is yes to any, please give details below.

AR received travel grants from Oxford Nanopore Technologies to attend scientific conferences. The sponsor had no role in the interpretation, or writing of the review or any scientific publications from AR.I declare that I have no other competing interests.

I agree to the open peer review policy of the journal. I understand that my name will be included on my report to the authors and, if the manuscript is accepted for publication, my named report including any attachments I upload will be posted on the website along with the authors' responses. I agree for my report to be made available under an Open Access Creative Commons CC-BY license (http://creativecommons.org/licenses/by/4.0/). I understand that any comments which I do not wish to be included in my named report can be included as confidential comments to the editors, which will not be published.

Choose an item.

To further support our reviewers, we have joined with Publons, where you can gain additional credit to further highlight your hard work (see: https://publons.com/journal/530/gigascience). On publication of this paper, your review will be automatically added to Publons, you can then choose whether or not to claim your Publons credit. I understand this statement.

Yes Choose an item.
